# Recent trends in therapeutic application of engineered blood purification materials for kidney disease

**DOI:** 10.1186/s40824-022-00250-0

**Published:** 2022-02-04

**Authors:** Cui Gao, Qian Zhang, Yi Yang, Yangyang Li, Weiqiang Lin

**Affiliations:** 1 Kidney Disease Center, The First Affiliated Hospital, Zhejiang University School of Medicine, Hangzhou, 310003 Zhejiang China; 2 Department of Nephology, The Fourth Affiliated Hospital, Zhejiang University School of Medicine, Yiwu, 322000 Zhejiang China; 3 International Institutes of Medicine, The Fourth Affiliated Hospital, Zhejiang University School of Medicine, Yiwu, 322000 Zhejiang China; 4 Key Laboratory of Women’s Reproductive Health Research of Zhejiang Province, Women’s Hospital, Zhejiang University School of Medicine, Hangzhou, 310006 Zhejiang China; 5 Cancer Center, Zhejiang University, Hangzhou, 310058 Zhejiang China

**Keywords:** Blood purification, Renal replacement therapy, Engineered biomaterials, Adsorbent, Polymeric composite membrane

## Abstract

Blood purification is a commonly used method to remove excess metabolic waste in the blood in renal replacement therapy. The sufficient removal of these toxins from blood can reduce complications and improve survival lifetime in dialysis patients. However, the current biological blood purification materials in clinical practice are not ideal, where there is an unmet need for producing novel materials that have better biocompatibility, reduced toxicity, and, in particular, more efficient toxin clearance rates and a lower cost of production. Given this, this review has carefully summarized newly developed engineered different structural biomedical materials for blood purification in terms of types and structure characteristics of blood purification materials, the production process, as well as interfacial chemical adsorption properties or mechanisms. This study may provide a valuable reference for fabricating a user-friendly purification device that is more suitable for clinical blood purification applications in dialysis patients.

**Graphical Abstract:**

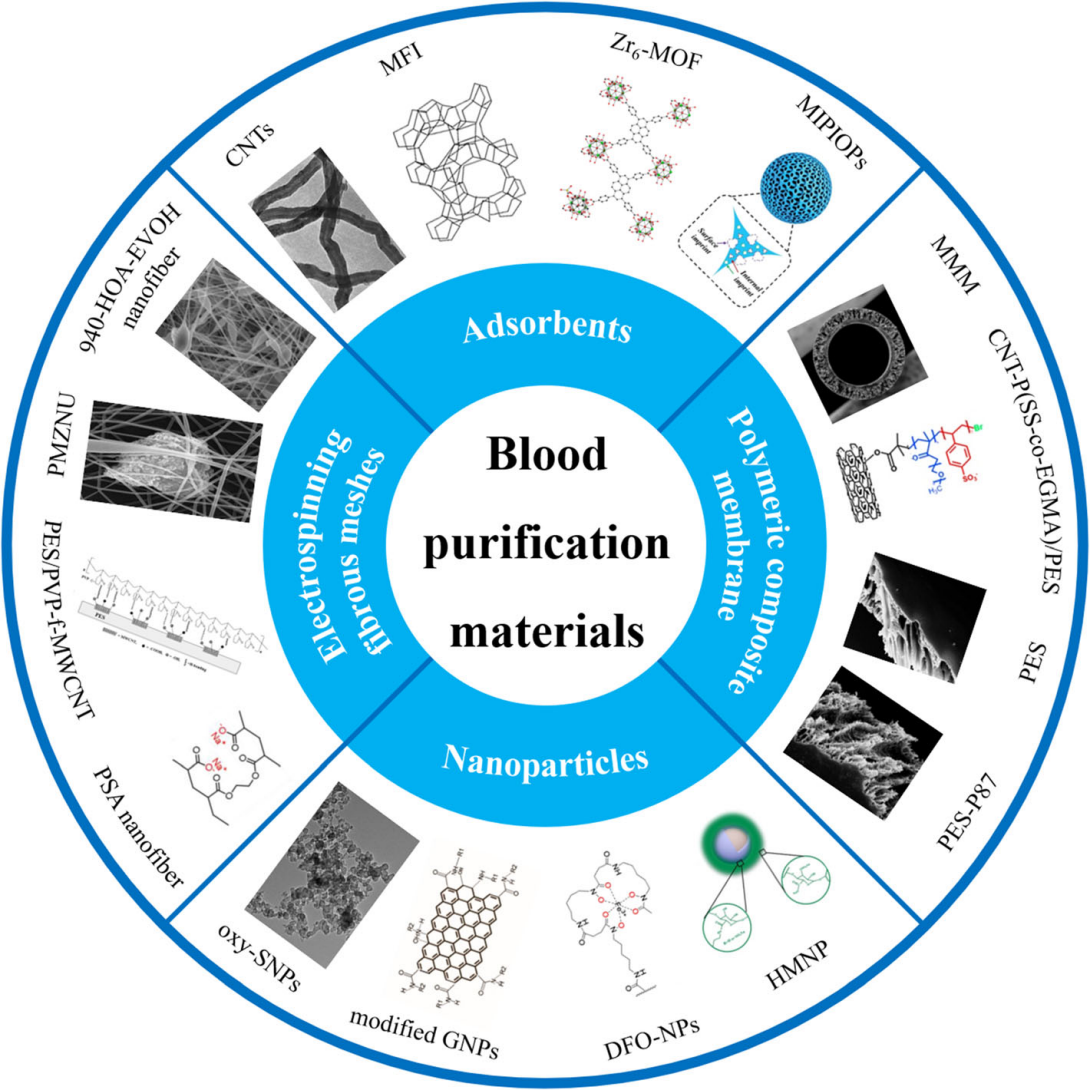

## Introduction

Uremic toxins often accumulate in patients with compromised kidney function, including those with end stage kidney disease (ESKD). This leads to serious illness, with renal replacement therapies as the only solution for survival. Many studies found reduced mortality and reports of a better quality of life among kidney recipients; however, demand outstrips availability, where only 25% of ESKD patients receive a kidney [1]. Worldwide, it is estimated that about 280 patients per million undergo regular hemodialysis or peritoneal dialysis treatment, while five-year survival of these people is between 13% and 60% lower than people in the general population of similar ages [1–3]. Owing to population aging, as well as increased prevalence of diabetes and hypertension, the incidence of ESKD is expected to rise over the next decades [4]. What’s more, the accumulation of uremic retention solutes at high concentration is associated with adverse outcomes in dialysis patients, including high mortality and low overall health related quality of life [5–8].

**Fig. 1:**
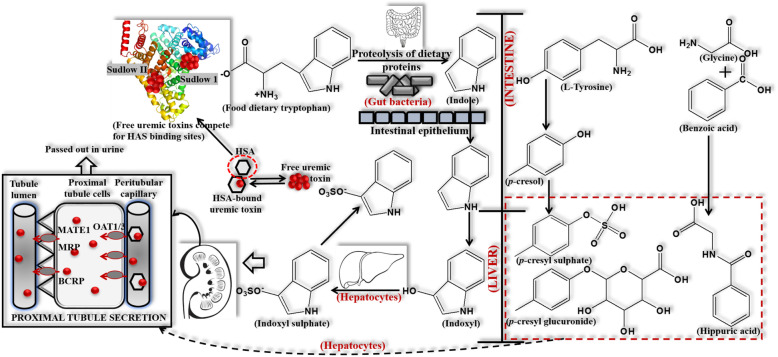
Pathway for production and clearance of protein-bound indoxyl sulfate (IS), p-cresyl sulphate (PCS), p-cresyl glucuronide (pCG) and hippuric acid (HA) toxins from the body. Reproduced with permission [16]. Copyright 2021, Elsevier

**Fig. 2:**
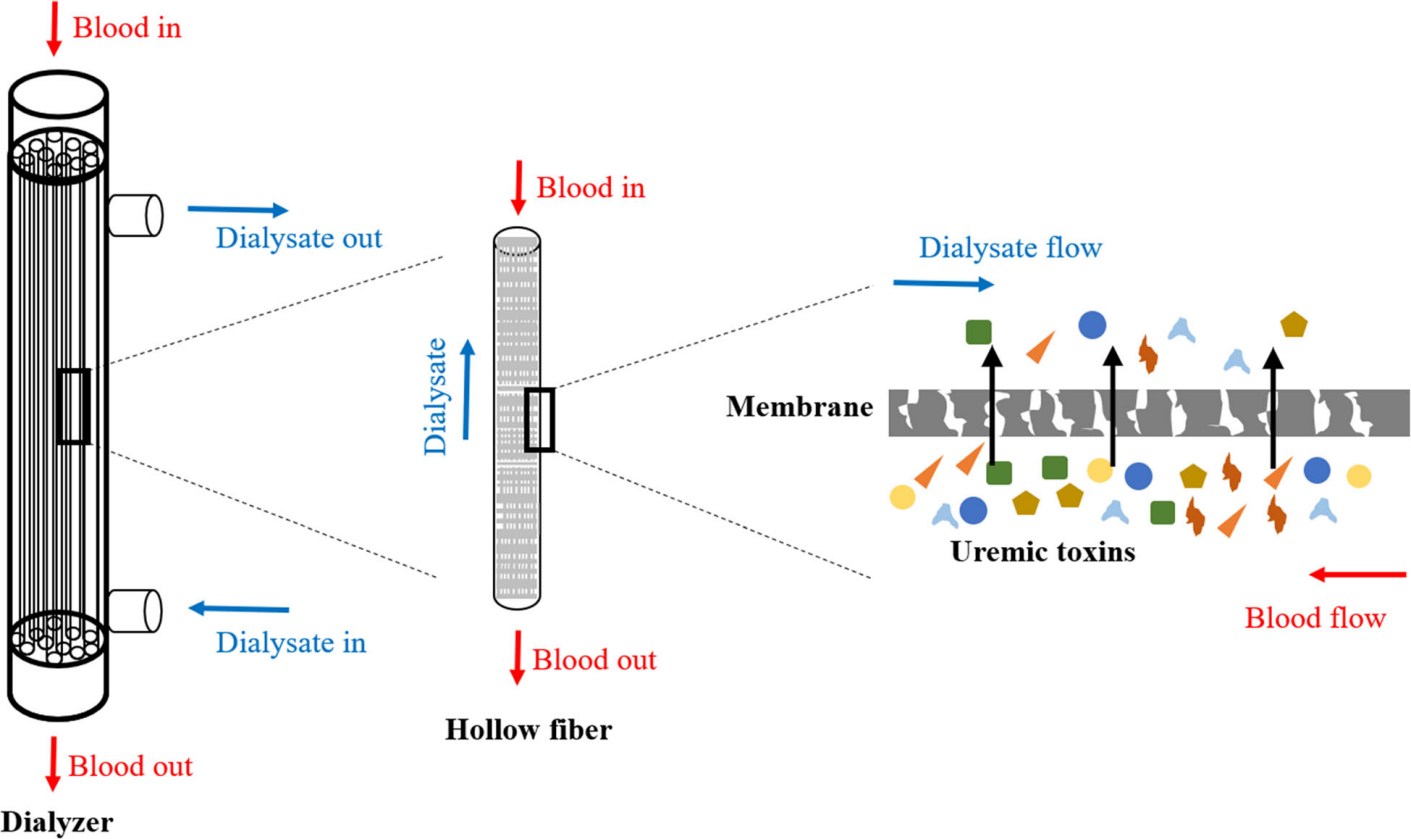
The basic mechanism of hemodialysis

Creatinine is a major uremic toxin, and its assemblage in blood causes a series of toxic symptoms that can reduce kidney function and consequently accelerate renal decline [9]. Protein-bound uremic toxins (PBUTs) are small molecules that primarily bind to the transport protein, human serum albumin in blood [10], and are involved in the generation of reactive oxygen species (ROS) [11]. While PBUTs are especially known to be associated with adverse/toxic effects, including cardiovascular disease [12–14], progression of kidney failure [15] and mortality [5], their renal clearance mechanisms and roles in uremic pathophysiology remain unclear [16]. Frequently, p-cresyl sulfate (PCS), indoxyl sulfate (IS), 3-Carboxy-4-methyl-5-propyl-2-furanpropionic acid (CMPF) and indole-3-acetic acid (IAA) are the most discriminating biomarkers of uremia, and are considered to be prototype protein bound uremic toxins that can bind more than 90% of plasma proteins. All four of these PBUTs have an aromatic ring and ionic functional group, and can form non-covalent bonds, such as Van der Waals forces and hydrogen bonds, as well as electrostatic and hydrophobic interactions [16]. With PBUTs, there is a balance between both protein-bound and unbound forms in their secretion and circulation, and the inherent clearance of unbound toxins is largely dependent on renal tubular secretion via specific basolateral organic anion transporters (OATs). The pathway for the production and clearance of protein PBUTs is shown in Fig. 1 [16]. Uremic toxicity is linked to endothelial dysfunction and immune dysfunction, which causes inflammation and activation of innate immune effectors through the induction of a pro-inflammatory state that involves Toll-like receptors and inflammatory cytokines [14]. Thus, the sufficient removal of these toxins from blood increases the efficacy of dialysis, which in turn increases the survival rate in ESKD patients. The basic mechanism of hemodialysis is shown in Fig. 2. However, the elimination of excess metabolites using conventional extracorporeal renal replacement therapies via semipermeable porous polymeric membranes has not currently been effective in clinical use. In particular, the PBUTs are difficult to remove through hemodialysis because each of these molecules possesses an aromatic moiety and ionic functional group that allows for binding to several adsorption sites on human serum albumin [17–20]. It has been reported that less than 35% reduction ratio of both PCS and IS are eliminated during high-flux hemodialysis [11].

In view of hemodialysis as a life-sustaining extracorporeal treatment for kidney failure, where large numbers of ESKD patients need long-term dialysis, it is necessary to prepare materials with simple and low-cost fabrication methods. Although the efficiency and selectivity properties of dialysis membranes have improved in recent decades, the traditional dialysis treatment remains an inconvenient, time consuming and expensive process [21–23]. Recently, several biomedical blood purification materials have been developed to increase the toxin removal rate, some of which also possess characteristics like less toxicity and low production cost. In this article, we summarize these results by category in order to offer information for further research about dialysis materials with improved properties.

## Biomedical materials for toxin removal

### Adsorbents

**Fig. 3:**
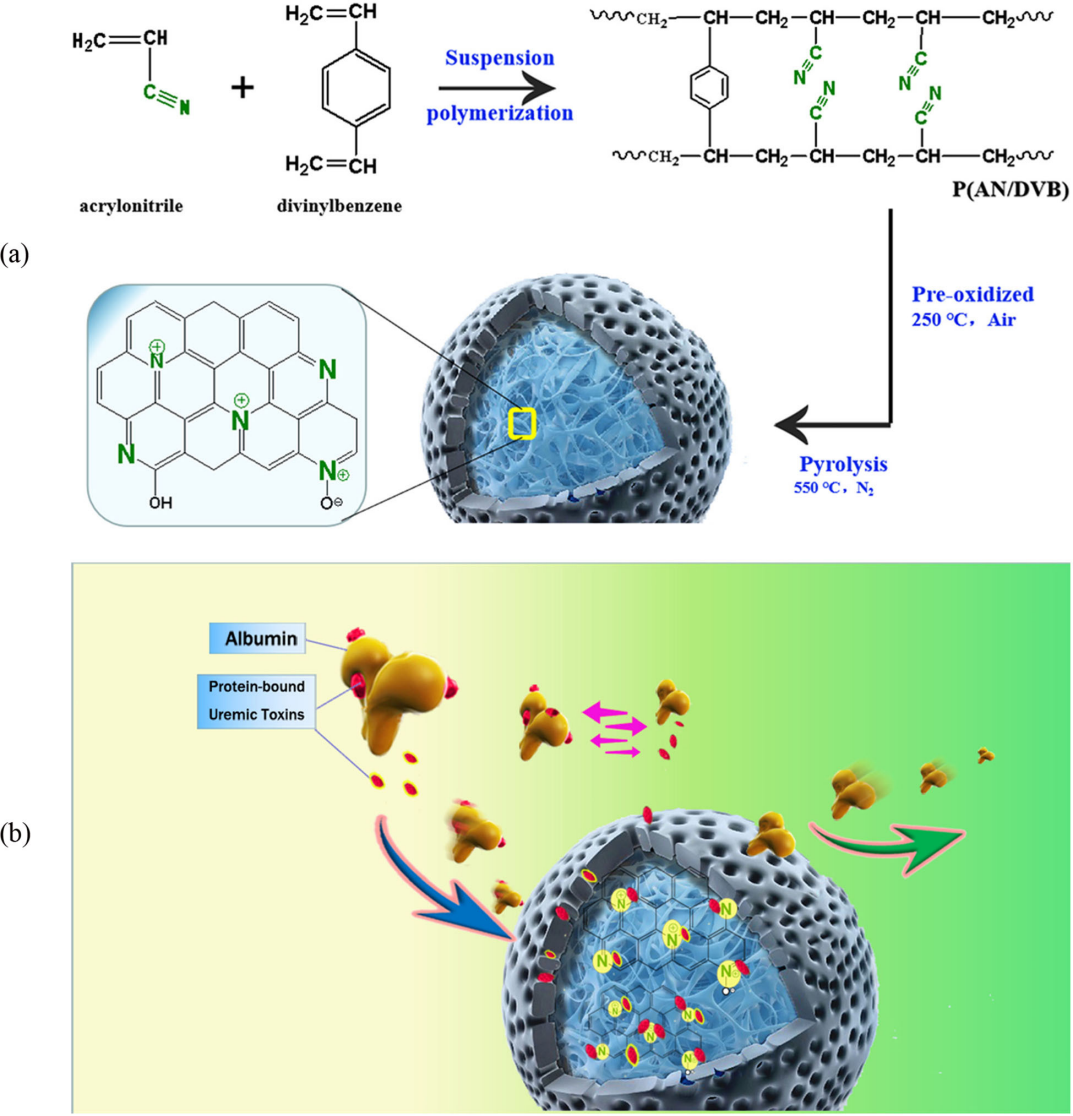
**a** Schematic illustration of the preparation of NPCA beads. **b** Illustration of the removal mechanism of PBUTs by using NPCA. The symbol ’N’ in the scheme represents the richest type of N atoms, which are assigned to be pyridinic N, pyridonic/pyrrolic N and quaternary N in NPCA. Reproduced with permission [31]. Copyright 2021, Elsevier

Activated carbon (AC) and zeolite are two common adsorbents applied in purification to increase ultrafiltration properties. AC has a long track record in detoxification systems as an adsorptive particle because it can adsorb a broad range of solutes [24–26]. It possesses a vast pore size but is not size selective. AC exhibits a high adsorption capacity of uremic toxins, but it also simultaneously removes other useful molecules. Reducing the diameter of a particle is a feasible way to raise outer surface area and thus increase accessible active sites and fast binding. However, the application of suspensions with powdered activated carbon requires a membrane filter to keep the sorbents suspended and prevent any particle contact with blood, which usually limits the amount of sorbent volume and inevitably results in lower concentrations of sorbents [27]. Compared with AC, carbon nanotubes (CNTs) possess higher surface area, larger aspect ratio and better adsorption performance for uremic toxins, and thus they are considered as the more suitable material for the design of a high efficient blood purification membrane [28–30]. Liu et al. fabricated nitrogen-containing porous carbon adsorbent (NPCA) beads that had added advantages in terms of biosafe, effective clearance of PBUTs and possessed satisfactory in vitro hemocompatibility. NPCA were prepared via pyrolying a cross-linked porous acrylonitrile/divinylbenzene copolymer beads (Fig. 3a) [31]. The NPCA beads showed the higher adsorption rates of PBUTs (IS, PCS and IAA; 45%, 44% and 95%) and an equivalent adsorption performance towards the middle-molecular-weight toxins (PTH and IL-6) in human plasma compared with HA-130/MG-150 (a commercial adsorbent used in clinic). The PBUTs removal mechanism of NPCA is ascribed to competition between nitrogen functional groups on NPCA and proteins for PBUTs binding via the electrostatic interactions, and it has no strong relationship with the pore structure (Fig. 3b) [31].

Unlike amorphous AC, crystalline adsorbents can allow for direct structural characterization that is able to assist in understanding the interactions between an adsorbent and a toxin, which is critical for the design of superior adsorbent materials. Zeolite is one of the best alum inosilicates applied for numerous molecular sieves and possesses high resistance in chemical and thermal processes [32]. Zeolites are non-toxic, stable in aqueous solution and not degraded under physiological conditions. Moreover, different types of microporous zeolite have channel systems in different sizes that can selectively adsorb some uremic toxins [33–36], and they can be found naturally or produced synthetically. These features make zeolites a potential material for artificial kidney applications. The size and shape of zeolite particles are important for creatinine uptake ability when incorporated inside the membrane [37, 38]. For instance, microparticles have better performance on creatinine adsorption than nanoparticles. Meanwhile, compared with rod nanoparticles, spherical nanoparticles are a better choice to incorporate into the electrospinning polymer fibers for improving creatinine clearance rate [37].

**Fig. 4:**
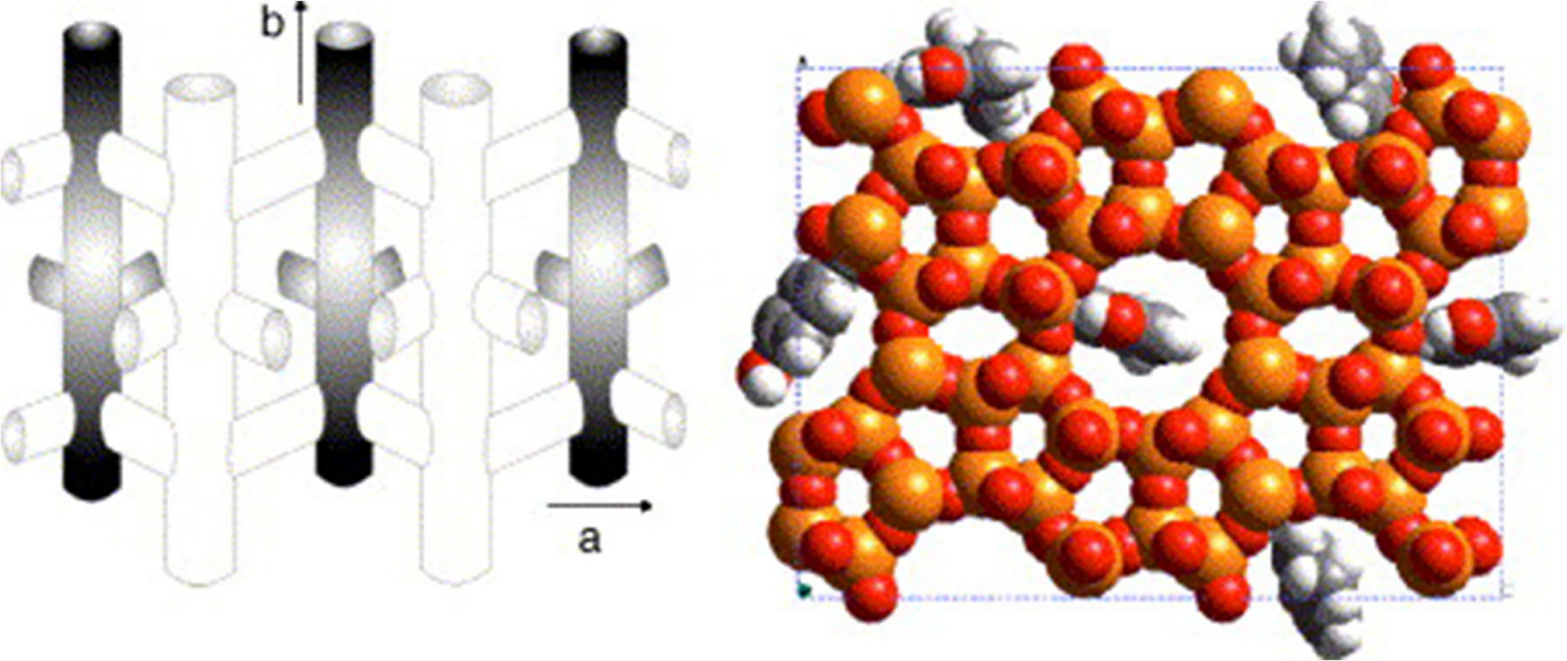
**a** Channel system of zeolite silicalite and **b** Monte Carlo simulation of p-cresol adsorption onto silicalite with view into b-direction. Reproduced with permission [39]. Copyright 2006, Elsevier

Wernet et al. investigated the elimination of uremic toxins using zeolites of different structural types [33]. They concluded that the adsorption properties of zeolites not only depend on the size of the channels, but also on the interactions between the adsorbates and the zeolite lattices. More specifically, zeolite silicalite (MFI) demonstrates strong adsorption of p-cresol (about 60% p-cresol in solution with concentrations close to those found in uremic patients), which is attributed to channel opening/size effect and hydrogen bonding interactions. The mechanisms of adsorption are shown in Fig. 4 (e.g., the adsorption of p-cresol onto silicalite zeolite) [39]. MFI possesses less equilibration time and a higher adsorption level of p-cresol than cellulose-based membranes and synthetic membranes [39]. Besides, it is possible to selectively eliminate 75% of creatinine in solution by an acidic mordenite (MOR), which is basically due to electrostatic interactions between the O-functional group and the Brønstedt sites present in the MOR pores [33]. The high adsorption of uric acid on ion-exchanged stilbites (STIs), such as Ca-STI, K-STI and Na-STI, can be attributed to the electrostatic interaction between a cation and the negative atom of a polar molecular bond. However, there is a potential way to increase the adsorption properties of STI through the formation of strong covalent interactions using cations with an affinity for uremic toxins [33]. Bergé-Lefranc et al. further demonstrated that pure silica MFI possesses a better capacity for the removal of p-cresol than aluminosilicate MFIs (Si/Al=30, compensating charges with H^+^, Na^+^, K^+^ and Mg^2+^) [34]. They found that compared with in solution, p-cresol affinities for the zeolites are generally reduced in human serum (obtained from dialysis patients) because proteins obstructed the pore system. Thus, zeolites can only be employed in the ultra-filtrate without direct contact with serum albumin [35].

Metal-organic framework (MOF) is one kind of novel hybrid material, which has high thermal and chemical stability, and has been demonstrated to be more effective than AC or mesoporous silica materials, due to its ultrahigh porosity and active sites [40–42]. And BET surface area and internal MOF cage diameter have been previously hypothesized as key parameters in the adsorption capacity of MOFs [43]. MOF is made of metal ions and organic linkers through coordinatebounds to form a 1D, 2D or 3D structure [40, 44]. MOFs have quickly gained traction in applications for, but are not limited to, bioactive compound separation [45], water purification [46, 47], drug delivery [48] and gas separation [49]. Nevertheless, the usage of MOFs in artificial kidney applications is still in the early stages. Furthermore, MOFs possess exceptional tenability, and, unlike other classes of crystalline materials, they can be systematically studied and incorporated into a host of functionalities [50].

**Fig. 5:**
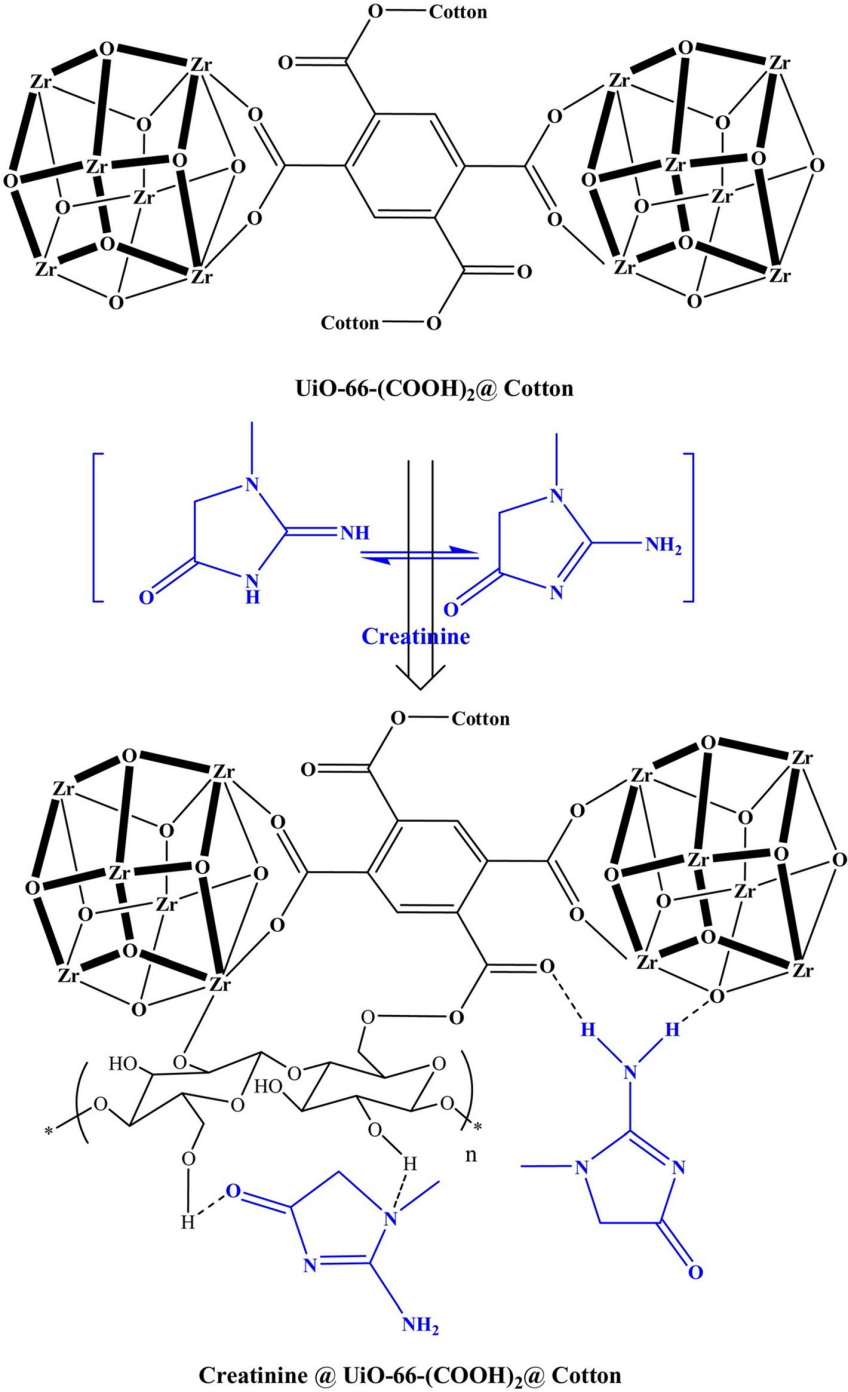
Intermolecular interactions between creatinine and UiO-66-(COOH)_2_@cotton composite. Reproduced with permission [51]. Copyright 2018, Elsevier

Abdelhameed et al. reported on one type of Zr-based MOF that was grown within cotton fabric composite, which can be regenerated by sonication using methanol, while its efficiency in creatinine removal only reduces by 16% (98% vs. 82%) after three generation cycles [51]. This in-situ composite was directly formatted without fabricating UiO-66-(COOH)_2_, and instead used 1,2,4,5-benzenetetracarboxylic dianhydride, zirconium tetrachloride and cotton fabric [51]. The UiO-66-(COOH)_2_@cotton fabric composite adsorbs creatinine through weak interactions between bonding sites of MOF and function groups of creatinine. The reaction mechanism is demonstrated in Fig. 5. Besides, it had been reported that by functionalizing UiO-66 with isovalent substituents such as -NH_2_, -OH, and SO_2_H considerably improved adsorption capacity by changing the electronic properties of MOF [52, 53]. Klaudia et al. synthesized the series of UiO-66 materials varying with final content of amino groups by changing the H_2_BDC/H_2_BDC-NH_2_ ratio and adding hydrochloric acid (HCl) during modulated synthesis [52]. They confirmed that UiO-66-NH_2_(75%) (with 75 mol% -amino groups) and UiO-66-NH_2_(75%)12.5%HCl performed better adsorption capacity for hippuric acid and 3-indoloacetic acid, revealing an analogous adsorption capacity to NU-1000, meanwhile, the prepared UiO-66-NH_2_ did not still showed any cytotoxic effect.

**Fig. 6:**
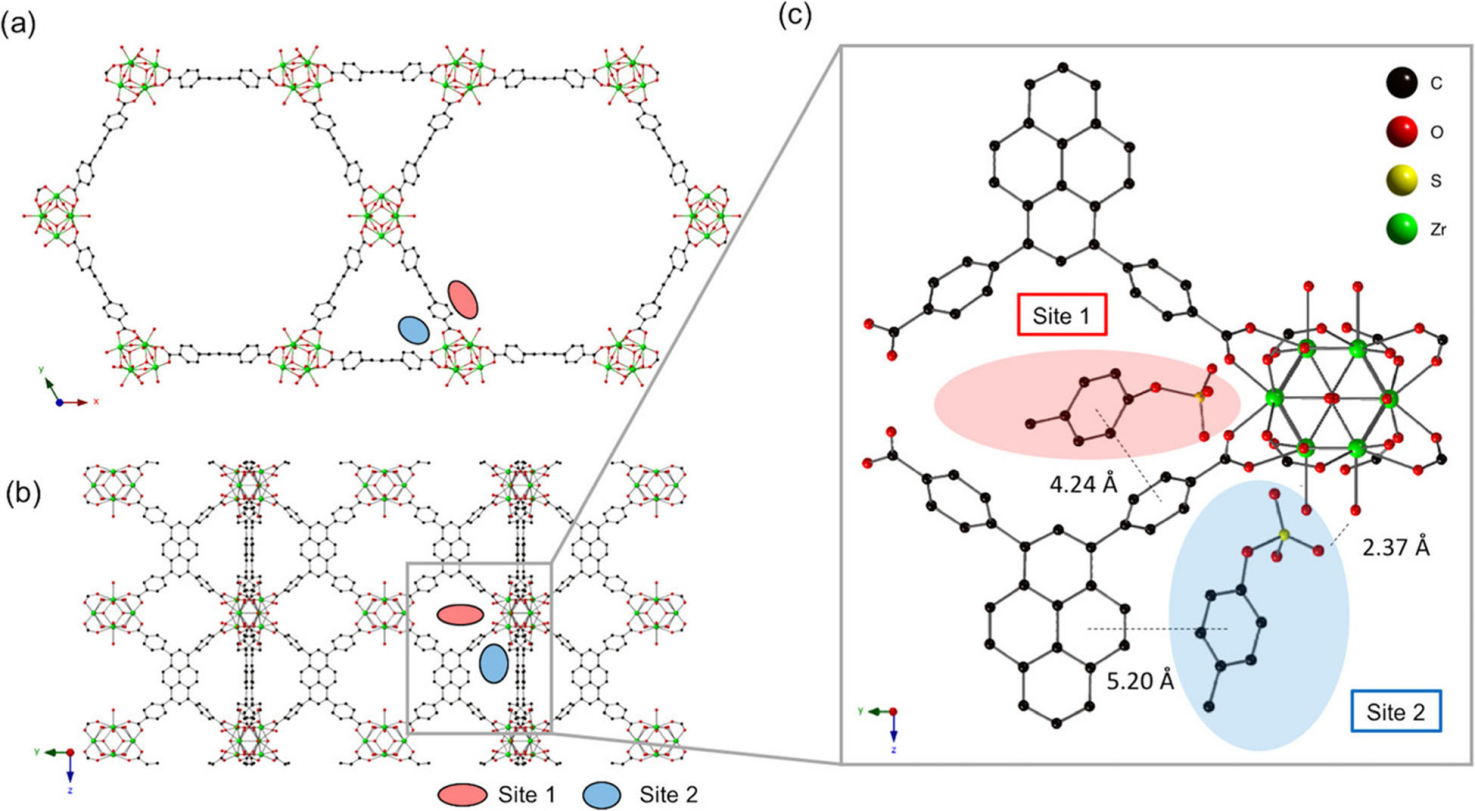
**a** Crystal structure of NU-1000 viewed down the c-axis. **b** Orthogonal view prior to p-cresyl sulfate exposure. **c** Optimized geometry of p-cresyl sulfate-pyrene and Zr_6_ node domains after p-cresyl sulfate adsorption. Reproduced with permission [50]. Copyright 2019, American Chemical Society

Kato et al. observed the adsorption behavior of p-cresyl sulfate in a series of Zr_6_-based MOFs with varying topology, connectivity and linker structure, including UiO-66, UiO-67, UiO-NDC, PCN-608-OH, NU-901, NU-1000, NU-1010, NU-1200 and MOF-808 [50]. These zirconium-based MOFs possess comparable surface areas and pore sizes. Among them, NU-1000 exhibits the highest toxin removal efficiency, where more than 70% of p-cresyl sulfate, 98% of indoxyl sulfate and hippuric acid in solution, as well as about 93% of p-cresyl sulfate can be removed from human serum albumin. This can be attributed to the highly hydrophobic adsorption sites that are sandwiched by two pyrene linkers, as well as the hydrogen bonding between the hydroxyl groups on the Zr_6_ nodes and the ionic functional groups of the adsorbates [50]. Two sites of p-cresyl sulfate on NU-1000 are depicted in Fig. 6. These two locations approximately have the same occupancy, where electrostatic interactions with hydroxyl groups on the Zr_6_ nodes and *π-π* interactions with pyrene-based linkers are important factors in adsorption ability. However, Cuchiaro et al. pointed out that application of NU-1000 is limited as it is commercially unavailable, and iron is a desirable alternative to zirconium [43]. Cuchiaro et al. synthesized MIL-100(Fe) and MOF-808, both of which have the same organic linker with MOF-808 but MIL-100(Fe) is less toxic due to its iron-based metal nodes [43]. They found p-cresyl sulfate uptake for MIL-100(Fe) was three times greater than that for MOF-808, which was less correlated to BET surface area, pore window size, cage diameter, and number of aromatic carbons in organic linker, indicating that metal-iron interactions maybe occurring more favorably in MIL-100(Fe) than MOF-808.

**Fig. 7:**
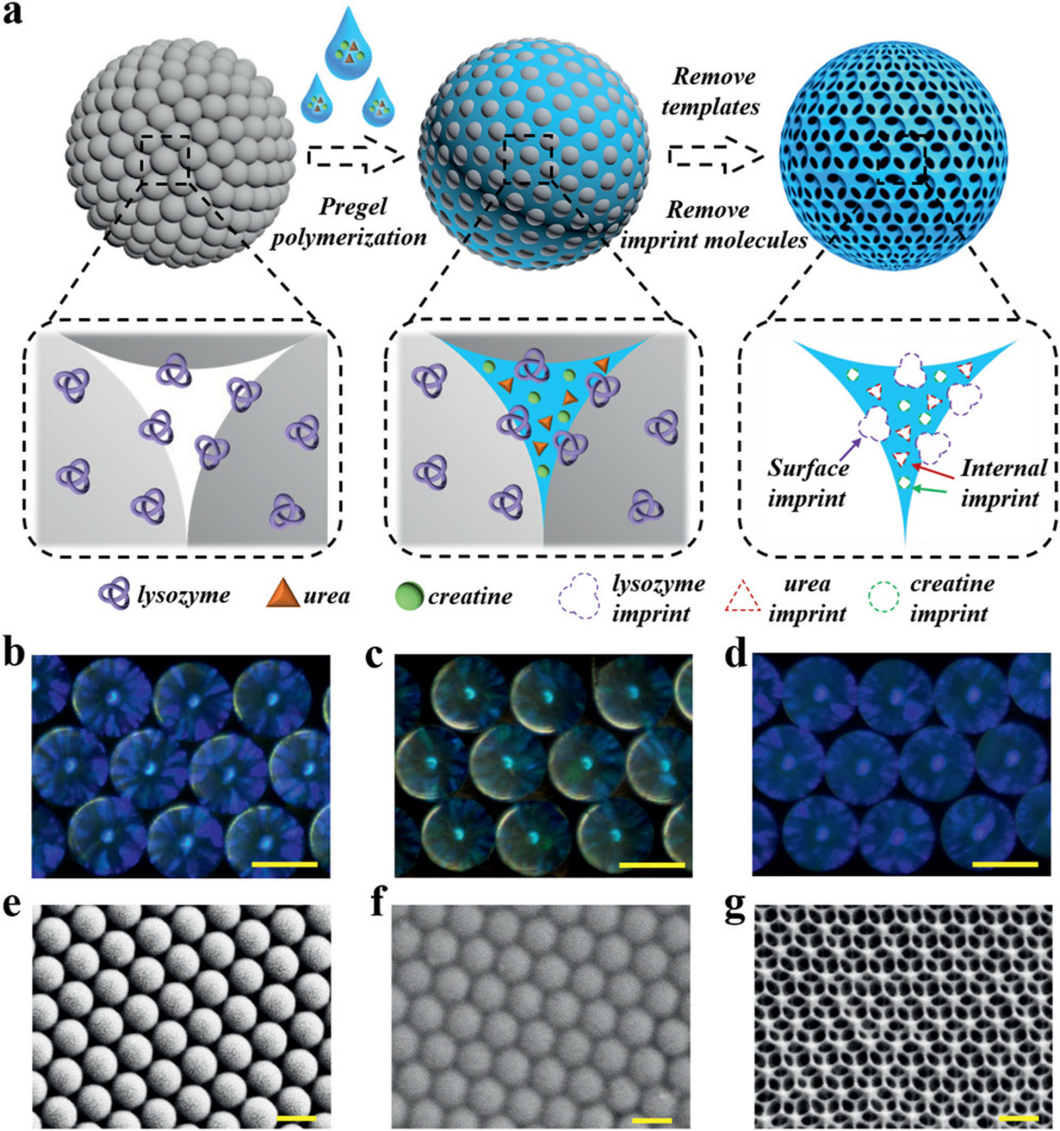
**a** Schematic diagram of the fabrication process for the combinative MIPIOPs. **b**–**d** Reflection microscopic images of the lysozyme-functionalized SCCBs (**b**), the molecular imprinting hydrogel hybrid SCCBs (**c**) and the MIPIOPs (**d**). **e**–**g** Scanning electron microscopy (SEM) images of the microstructures of lysozyme-functionalized SCCBs (**e**), the molecular imprinting hydrogel hybrid SCCBs (**f**) and the MIPIOPs (**g**). Scale bars are 250 μm in (**b**–**d**) and 200 nm in (**e**–**g**). Reproduced with permission [54]. Copyright 2020, Wiley

Inspired by the self-purification ability of the kidney, Chen et al. proposed a new adsorbent called molecular-imprinted polymer inverse opal particles (MIPIOPs) [54]. Here, the fluidic environment is used to resolve the dilemma of insufficient contact between adsorbent materials and targets molecules. The MIPIOPs are embedded in a microfluidic chip with a herringbone mixer and the scale can be readily amplified to accommodate a large number of MIPIOPs for purification. Moreover, the herringbone channels can generate chaotic advection of the fluid, and therefore improve the mixing and adsorption efficiency between the target biomolecules and MIPIOPs. The MIPIOPs are fabricated through a combinative imprinting process (Fig. 7). The silica colloidal crystal beads (SCCBs) were first fabricated, and then the silica nanoparticles at the surface of the SCCBs adsorbed the lysozyme through electrostatic interaction. The following step fully filled the void spaces between the silica nanoparticles of the lysozyme-functionalized SCCBs using a pregel mixture of methacrylate gelatin (GelMA), polyethylene glycol diacrylate (PEGDA), urea and creatinine. Then, the SCCBs and the imprint molecules, including lysozyme, urea and creatinine, were removed from the pre-gel polymer. The resultant MIPIOPs possess multiple molecular binding sites for lysozyme at the surface, while the urea and creatinine are in the interior. The MIPIOPs have unique features and display good blood compatibility, and it has been demonstrated that their adsorption capacity is maintained stably after being reused for five times [54]. Moreover, due to highly ordered 3D porous structure, they are imparted with photonic band gap properties that allow for monitoring and self-reporting the state of adsorption.

**Fig. 8:**
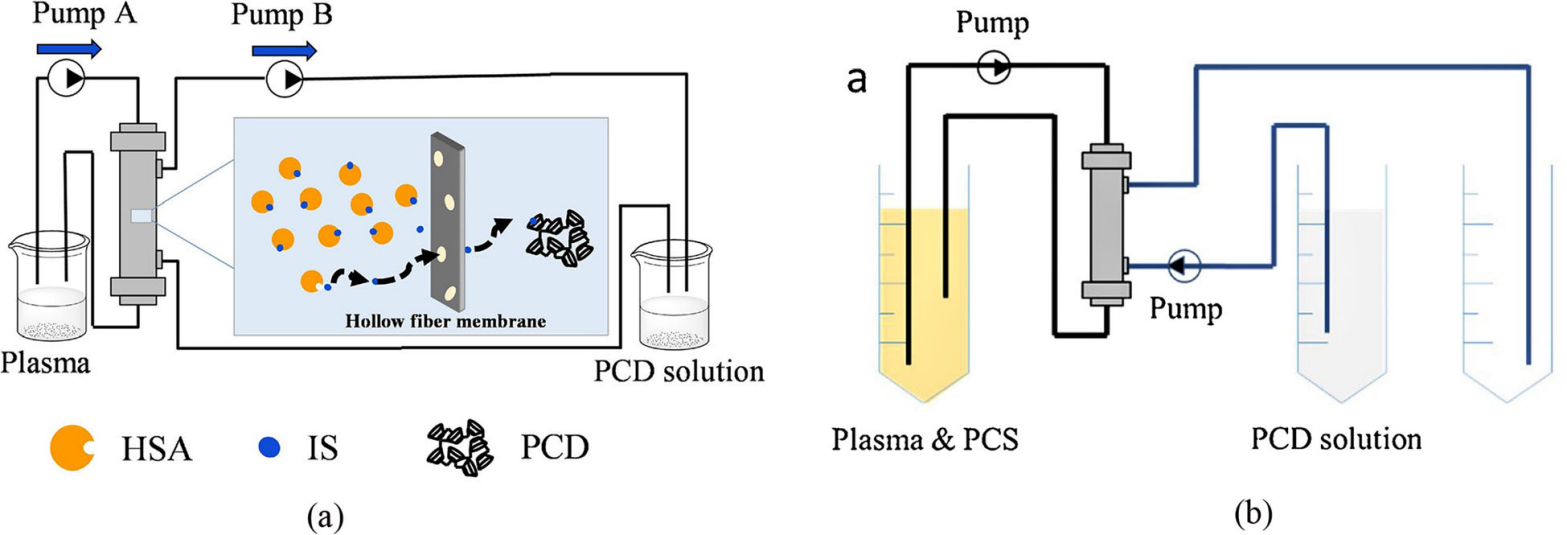
**a** Schematic representation of the two-compartment recirculation dialysis system with PCD as the adsorbent (a dialysate-recycled mode). Reproduced with permission [56]. Copyright 2018, Elsevier. **b** Scheme of a hemodialysis system in a once-through mode (the dialysate passed through hemodialysis system without recirculated usage). Reproduced with permission [57]. Copyright 2020, Elsevier

Cyclodextrins (CDs) are toroidal-shaped cyclic oligosaccharides composed of 6-8 D-glucose units (α, β, γ), with a hydrophilic exterior and a relatively hydrophobic inner cavity, which can encapsulate different low molecular weight lipophilic guests or macromolecules [55, 56]. The cup-shaped β-CD is composed of 7 glucose units, and is most commonly applied due to its sensitive recognition of diverse hydrophobic molecules by forming CD-guest complex. Li et al. proposed for the first time that adding crosslinked poly-β-cyclodextrins (PCD) into the dialysate of exterior dialyzer for improving mass-transfer efficiency of indoxyl sulfate (IS) (Fig. 8a) [56]. PCD was synthesized by a cross-link between β-CD and epichlorohydrin (ECH). β-CD firstly dissolved in NaOH solution, then ECH was added into the mixture, which was subsequently stirred at 30 °C for 2 h. According to the original study, the maximum binding capacity of PCD for IS about 45 mg g^-1^ and a 21% increase in removal rate has been achieved in the simulated dialysis experiment with introduction of PCD. The mechanism of IS binding to PCD may be that the indole ring of IS is accommodated in the cavity of β-CD through hydrophobic interaction and hydrogen bonding [56]. This new strategy exerts no negative impact on dialysis membrane, and is safe for clinical application since PCD has been proved with a low hemolysis rate and could not traverse the membrane to contact with the blood (hydrodynamic diameter of polymer is 9nm). Li et al. further compared adsorption capacity of poly-α-cyclodextrins, poly-β-cyclodextrins and poly-γ-cyclodextrins, and poly-β-cyclodextrins performed best with the maximum p-cresol sulfate (PCS) binding capacity (263 mg g^-1^) [57]. They found that the clearance of PCS in plasma through once-through mode (Fig. 8b) is more effective than recycled mode (96% vs. 43%), which attributed to the PCS concentration difference between plasma and dialysate increase with the removal of PCS. And the PCS (96%), hippuric acid (98%) and quinolinic acid (97%) in plasma were basically removed in PCD added dialysate system, further elucidating the broad-spectrum PBUT removal properties of PCD [57].

MXenes are a family of two-dimensional carbides and nitrides of transition metals with the general structure M_*n+1*_X_*n*_T_*x*_ (M is early transition metal, such as Ti, V, Nb, etc.; n+1=1-3; X is C and/or N; T_*x*_ represents the surface terminations, such as O, OH, F, and/or Cl) [58, 59]. MXenes have a unique combination of properties, including hydrophilic due to functionalized surfaces and stable colloidal solutions in water owing to high negative zeta-potential, and have been extensively researched in the biomedical field in recent years [58]. Ti_3_C_2_T_*x*_ is the first reported and most studied member of MXenes and is known to efficiently adsorb urea. And Ti_3_C_2_T_*x*_ has been strongly demonstrated to have relatively high biocompatibility and low biotoxicity in previous in vivo studies [60]. In the work of Zhao et al., Ti_3_C_2_T_*x*_ (Ti_3_C_2_-F, Ti_3_C_2_-O, Ti_3_C_2_-OH, fabricated from precursor Ti_3_AlC_2_ using 10 wt % hydrofluoric acid) was used as adsorbents in aqueous solution, and it performed the rapid adsorption rate and higher adsorption capacity towards creatinine and uric acid compared with conventional activated carbon [59]. The high affinity between Ti_3_C_2_T_*x*_ and creatinine in adsorption process is ascribed to hydrophilic surface terminations of Ti_3_C_2_T_*x*_ and intraparticle diffusion of creatinine between Ti_3_C_2_T_*x*_ layer. In the uric acid adsorption process, however, the high affinity might come from hydrogen bonding (Ti-OH … N) and van der Waals interactions. What’s more, considering that the Ti_3_C_2_T_*x*_ effectively adsorb urea, creatine and uric acid, as well as electrolyte cations (K^+^, Ca^2+^, Mg^2+^, etc.) can also occupy the active sites of Ti_3_C_2_T_*x*_, Zhao et al. proposed that Ti_3_C_2_T_*x*_ has the potential to be used as an efficient sorbent for the regeneration of dialysate. More recently, Wang et al. prepared Ti_3_C_2_T_*x*_ nanosheet by delaminating the etched Ti_3_C_2_T_*x*_ in deaerated water by ultrasonication, and discovered for the first time that ultrahigh removal capability of Ti_3_C_2_T_*x*_ towards IL-6, which demonstrated 13.4 times than that of traditional activated carbon and much faster removal rate [61]. The main mechanism for adsorption is the formation of hydrogen bonding between MXene and IL-6 (TI-X … H-N-C=O), as well as the immobilization of IL-6 on the surface of MXene nanosheets.

### Polymeric composite membrane

With the development of biomaterials science, various artificial polymers materials have gained intense popularity due to their easy access and good process ability. Thus, polymeric composites have been used in the fabrication of dialysis membranes [62–64]. For instance, polyethersulfone (PES) membrane can be blended with polyvinylpyrollidone (PVP) or polyacrylonitrile (PAN). Up to now, there have been many studies about the fabrication and modification of dialysis membranes using PAN, PES or PES/PVP as a primary matrix. Lu et al. demonstrated that 940-zeolite powders exhibit the best creatinine removal ability among four types of zeolites, including 940-HOA (bate), 840-NHA (ZSM-5), 500-KOA (L) and 720-KOA (Ferrierite). Meanwhile, they found that 940-PAN has higher creatinine adsorption capacity than 940-zeolites powders [65]. Additionally, they chose P87 to make zeolite-PES membranes through a spin-coating process. PES-P87 can adsorb 550 μg indoxyl sulfate per g membrane in deionized water and the mechanism is probably due to electrostatic attraction [66].

**Fig. 9:**
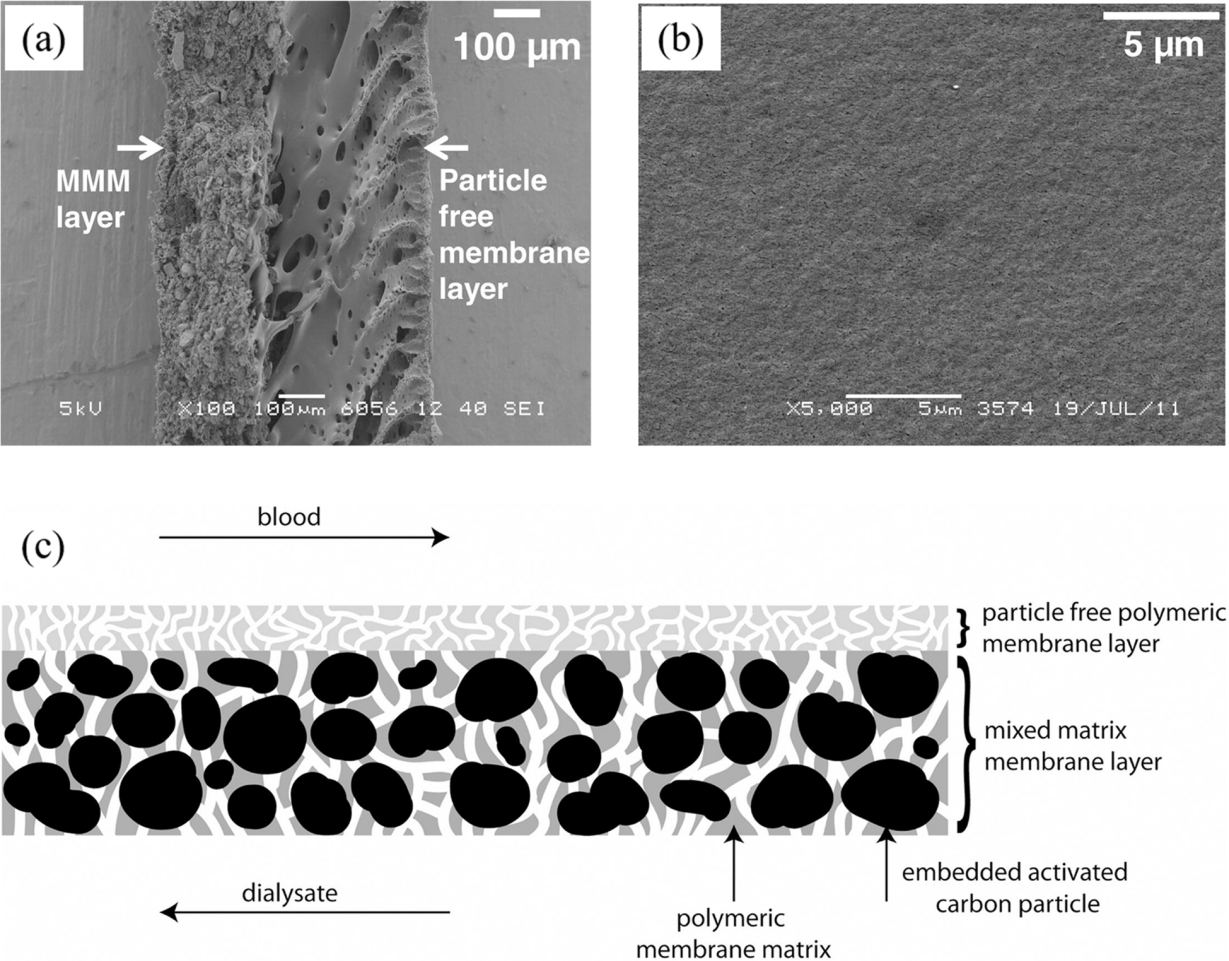
**a** SEM pictures of cross-sections of a dual-layer MMM. **b** Surface area pictures and SEM pictures of a dual-layer MMM. **c** Concept of dual-layer mixed-matrix membranes for blood purification. Reproduced with permission [67]. Copyright 2012, Elsevier

**Fig. 10:**
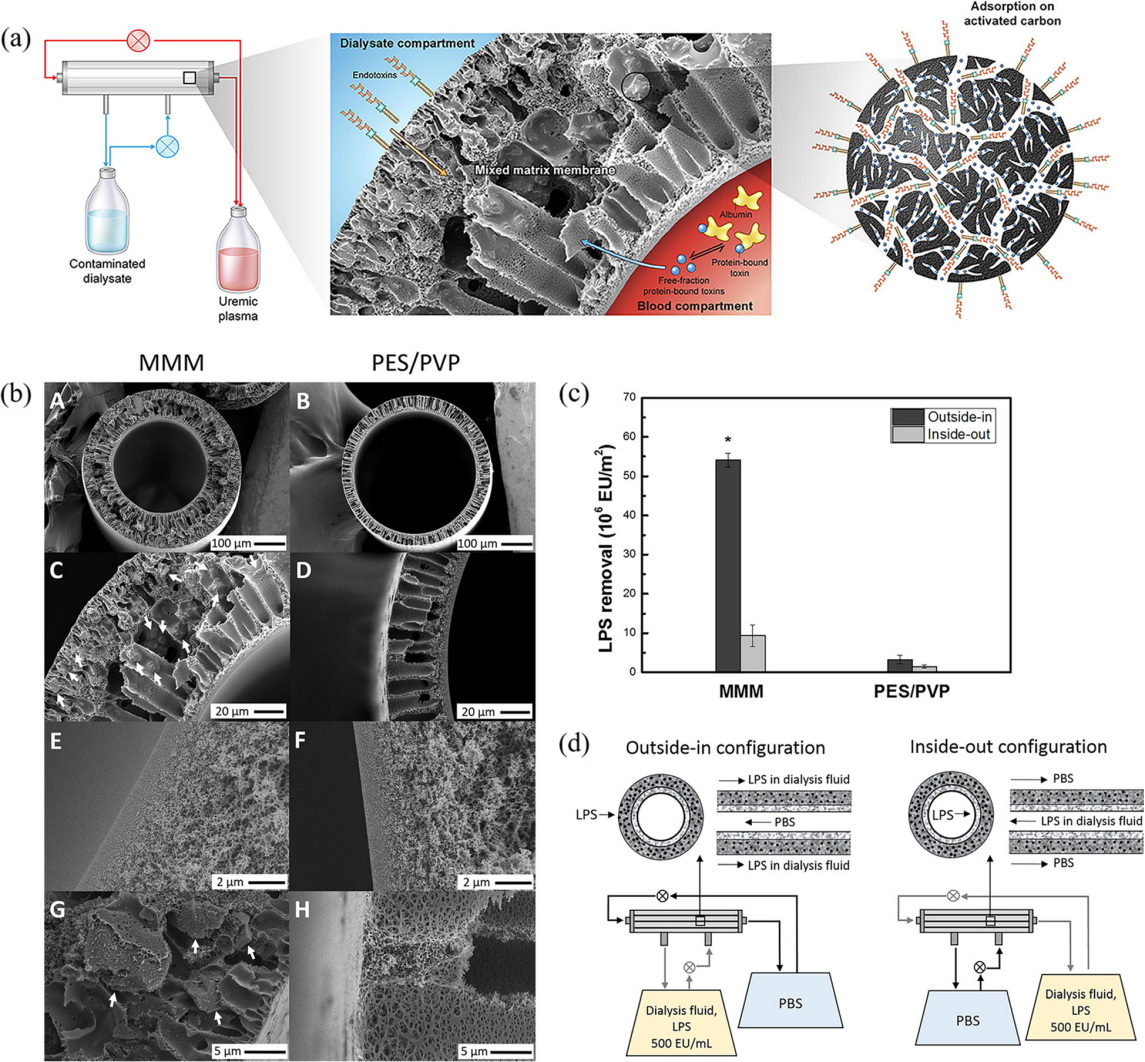
**a** Schematic of the matrix hemodialysis membrane for achieving endotoxin-free dialysate in combination with high removal of uremic toxins from human plasma. **b** SEM images of the dual layer MMM and PES/PVP HF. **c** Removal of LPS from the dialysate using the dual layer MMM and PES/PVP HF in a cross-flow diffusion experiment (performed in duplicate) after 4 h. **d** Experimental set-ups used for LPS dynamic adsorption experiments in outside-in configuration (left) and inside-out configuration (right). Reproduced with permission [71]. Copyright 2019, Elsevier

Tijink et al. were the first to propose a novel membrane with embedded adsorptive particles membranes called mixed-matrix membranes (MMMs), which can combine diffusion and adsorption in a single step [67], and exhibits high clean-water performance. Moreover, it can adequately remove both creatinine and protein bound uremic toxins from human plasma solutions [67, 68]. This dual layer MMM consists of a particle-free inner layer attached to the macro-porous layer that is composed of (AC) particles embedded in a PES/PVP matrix (Fig. 9). The inner particle-free layer is introduced on the blood-contacting side of the membrane to prevent particle release and improve hemocompatibility, which is revealed by lack of hemolysis, as well as low thrombin-antithrombin III-complex (TAT) and complement activation. However, the new MMMs have been shown to lower the relative concentrations significantly less (*p*<0.05) than AC particles in the case of hippuric acid, indoxyl sulfate and *p*-cresyl sulfate [67, 68]. Because the first generation MMMs were rather large in diameter, their mass transport characteristics needed improvement. Pavlenko et al. developed a new generation of MMMs with optimized characteristics [69]. Their strategy involved the use of a newly created spinneret in the dry-wet spinning procedure to obtain hollow fibers with smaller dimensions and tuned spinning conditions to optimize the loading of activated carbon particles and morphological characteristics. These second generation MMMs possess a smaller diameter, low ultrafiltration coefficient and no albumin leakage. More importantly, second generation MMMs have been demonstrated to have superior abilities in removing IS and PCS in comparison to the first MMMs (30 and 125%, respectively). Gremial et al. further evaluated in detail the hemocompatibility of two generations of MMMs, following the norm ISO 10993-4, using fresh human blood in ex vivo experiments [70]. The second generation MMMs have hemocompatibility profile similar to that of membranes currently applied in the clinic (Polysulfone® F60 as negative control and Cuprophan® F1 as positive control), but have good performance compared to the first generation MMM (one with high flux but some albumin leakage). This is mainly due to the high amount of PVP and the smoothness of blood contacting surface. Recently, Geremia et al. investigated an application of MMM for achieving endotoxin-free dialysate along with simultaneous high removal of uremic toxins with a single membrane (Fig. 10) [71]. The safety-barrier properties of MMMs in preventing transfer of pyrogens to the plasma have been revealed by analyzing inflammation in THP-1 monocytes incubated with dialysate that was contaminated by bacteria. Since the accessibility of AC to endotoxin is not compromised, the MMM can remove approximately 10 times more endotoxins (e.g., LPS from *E. coli* and *P. aeruginosa*) from dialysate than the PES/PVP HF membrane. Moreover, it can remove PBUTs from human plasma, including HA and IS without compromising AC adsorption capacity.

**Fig. 11:**
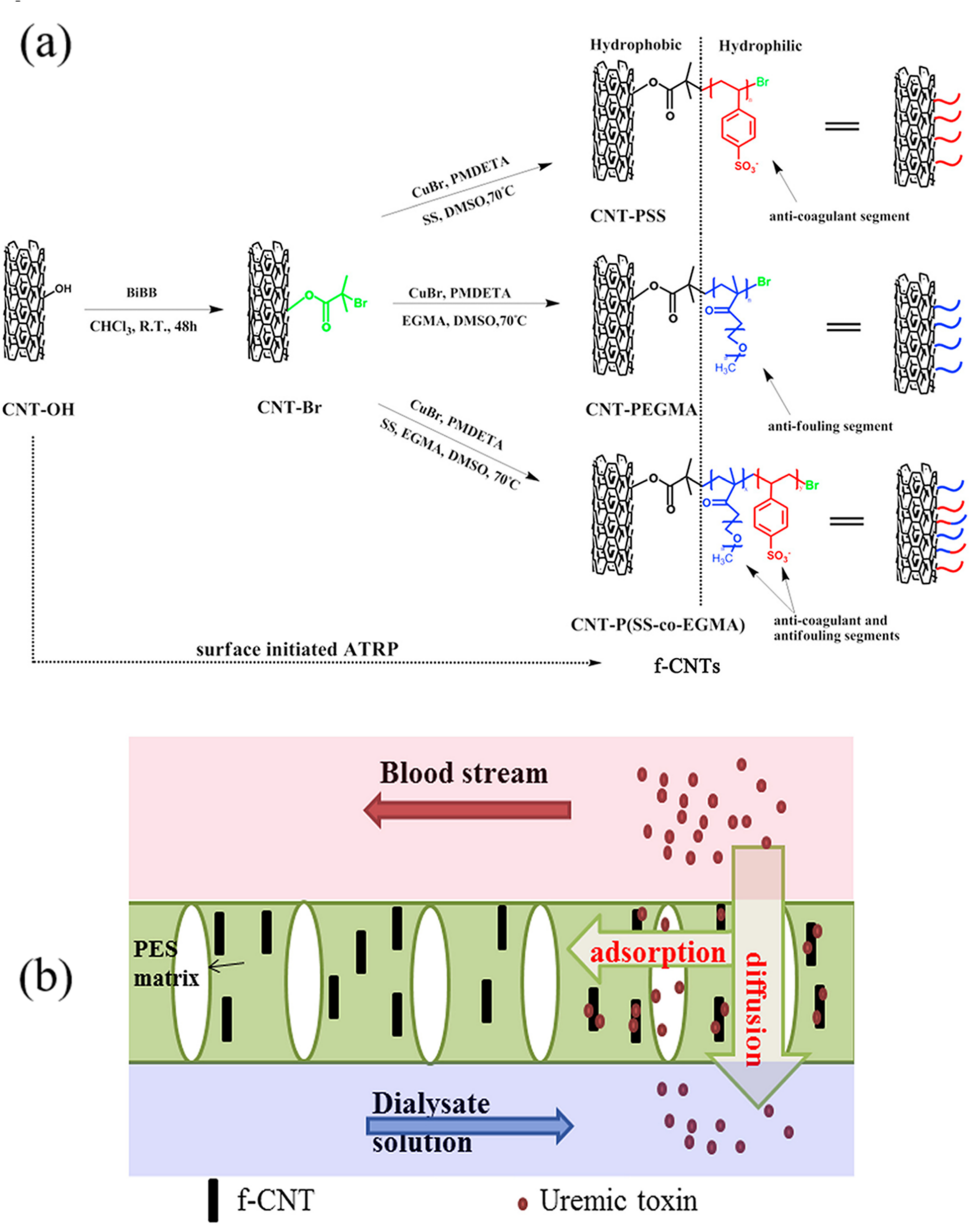
**a** Synthesis of heparin-mimicking polymer functionalized carbon nanotubes via surface initiated atom transfer radical polymerization. **b** Metabolic waste removal mechanism of the designed f-CNT/PES composite membrane in hemodialysis. Reproduced with permission [72]. Copyright 2015, Elsevier

In addition to improving purification efficiency, optimizing biocompatibility is also an urgent issue. A novel kind of heparin-mimicking membrane with integrated high biocompatibility and efficient uremic toxins removal has been designed and fabricated by Nie et al. using a spin-coating method and a subsequent liquid-liquid phase separation technique [72]. The heparin-mimicking polymer brush consists of a sodium styrene sulfonate (SS) that has an anti-coagulant segment and poly (ethylene glycol) methyl ether methacrylate (EGMA), which would improve the water permeability and antifouling ability of PES membranes. This brush is then grafted onto multiwall CNTs (f-CNTs). The synthesis method and toxins removal mechanism are presented in Fig. 11. This novel CNT-P (SS-co-EGMA)/PES combines adsorption and diffusion in one process, and it has been found to be very stable, as no f-CNT is eluted from the PES matrix during filtration.

Improving the removal ability and biocompatibility of polymeric composite membrane through methods that either embed adsorbent particles in it or graft a heparin-mimicking polymer on it are promising. However, the manufacturing technology for this kind of biomedical material needs to be further developed. For instance, fabricating fibers of reasonable diameter to ensure as best as possible that adsorption ability of the adsorbent embedded in polymer is not compromised, while avoiding loss of essential substances in the blood. Another crucial issue is the stability of these additional components that are added in membranes. Ideally, these components would not be easily denatured or detached from the membrane because these conditions could then cause allergies, hemolysis or coagulation reactions.

### Nanomaterials based adsorbents

#### Electrospinning fibrous meshes

**Fig. 12:**
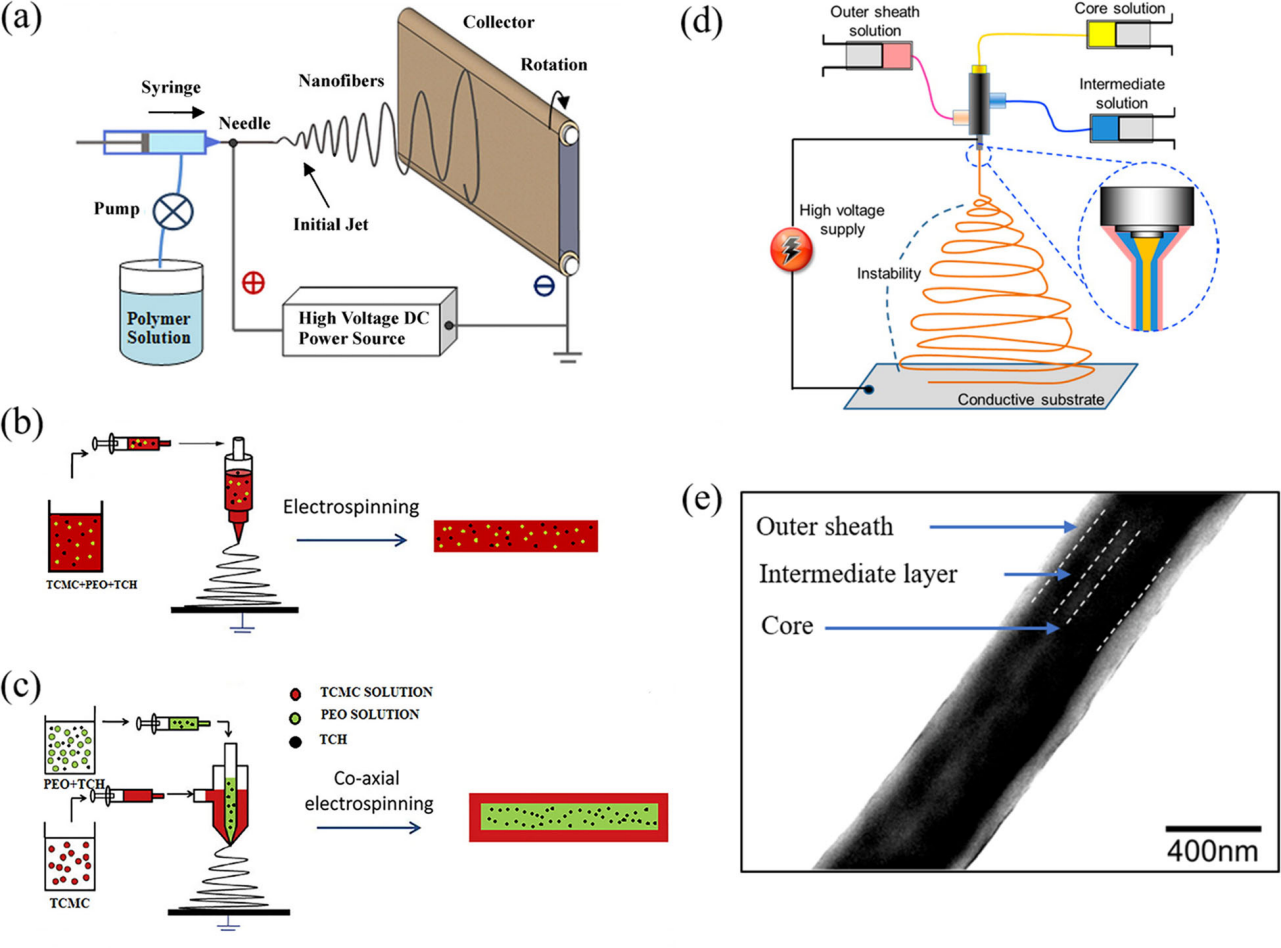
Schematic diagram of the electrospinning system for nanofibrous membrane fabrication. **a** General electrospinning system. Reproduced with permission [83]. Copyright 2010, Elsevier. **b** Hybrid electrospinning [84]. **c** Co-axial electrospinning [84]. Copyright 2017, Elsevier. **d** Tri-axial electrospinning [85]. **e** Transmission electron microscopy observation for tri-axial fiber with three-layered structure [85]. Copyright 2013, American Chemical Society

Nanomaterials have gained extensive attention as ideal adsorbents because of their large surface-to-volume ratio [73, 74]. Nanomaterials have huge potential for application in the field of biomedicine as innovative technology. Shrinking the diameter of polymer fibers can result in a much larger specific surface area, and, together with their functional groups, a proportionally larger ratio of exposed polymer chains [73]. Moreover, unlike conventional rigid porous structures, the macroscopic features of these polymeric nanofibers enable facile manipulation as a bulk matter, and the porous material made out of these nanofibers is a dynamic system, where the pore size and shape can be changed [74–77]. There are several methods of producing nanofibers, but the usefulness is limited by cost, production rate, material ranges selection and possible fiber assembly [74]. During the past few decades, polymeric nanofibers fabricated by electrospinning have gained popularity due to the versatility and cost-effectiveness of the fabrication method [73–75, 78]. Electrospinning employs electrostatic forces to produce fibers with a diameter from 50 nm to 2 μm by manipulating charged threads of polymer solutions. And it could fabricate functionalized nanofibers using hybrid or core-shell electrospinning (via the co-axial or tri-axial method) [79]. Generally, the hybrid method is used to functionalize nanofiber with drugs or enzymes (by mixing the polymer solution). While the co-axial and tri-axial electrospinning can be used to preserve unstable biological external particles and viruses, preventing the decomposition of unstable compounds, as well as achieving rapid and sustained drug release [80–82]. The schematic diagram of the electrospinning system for nanofibrous membrane fabrication is presented in Fig. 12. Almost any soluble polymer with sufficiently high molecular weight, such as natural polymers, polymer blends, nanoparticle- or drug-impregnated polymers and ceramic precursors, can be electrospun and yield a variety of continuous fibers with uniform diameters [74]. Furthermore, morphologies of fibers can be different, such as beaded, ribbon, porous and core-shell. Despite these possibilities, production conditions of composite nanofibers have to be carefully controlled during the whole process to avoid clogging of the needle tip [36].

**Fig. 13:**
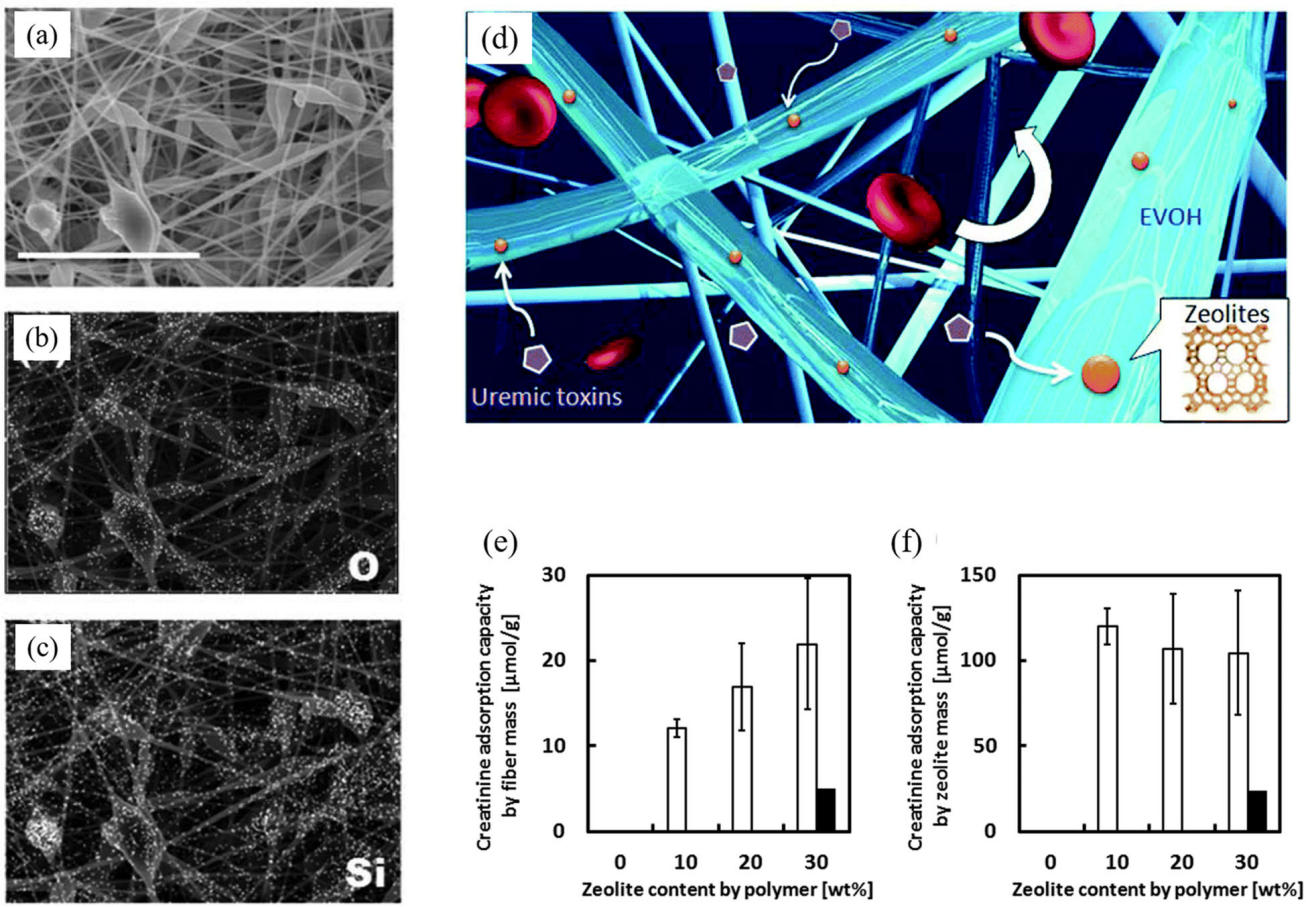
**a**–**c** SEM and energy dispersive X-ray spectroscopy (EDX) mapping images of beta-type 940-HOA zeolite (10 wt%)–EVOH nanofiber composite produced from 7 w/v% HFIP (scale bar: 8 μm). **d** Nanofiber is composed of blood compatible poly (ethylene-*co*-vinyl alcohol) as the primary matrix polymer and zeolites that are capable of selectively adsorbing uremic toxins. **e**, **f** Creatinine adsorption capacity of 940-HOA zeolite nanofibers by fiber mass (**e**) and zeolite mass (**f**) in the absence (open bar) or presence (closed bar) of flow. Reproduced with permission [36]. Copyright 2013, Royal Society of Chemistry

Nanekawa et al. developed a zeolite-polymer composite nanofiber mesh by electrospinning and used the blood compatible poly (ethylene-co-vinyl alcohol) (EVOH) as a primary matrix polymer (Fig. 13) [36]. They found that EVOH dissolved in 1,1,1,3,3,3-hexafluoroisopropanol (HFIP) can be electrospun very easily, over many days and produces more consistent fibers than when in isopropanol-water [36]. With the latter, they found EVOH can only be electrospun for a couple of hours, which then causes occasional clogging. The best base polymer solution is 7 w/v% EVOH in HFIP. For zeolites, 840-HOA (ZSM-5) and 940-HOA zeolites (beta type) have better creatinine adsorption capacity than 720-KOA (Ferrite) and 980-HOA (beta) when embedded in EVOH based fibers, while 940-HOA has a higher reusability. The EVOH matrix prevents the zeolites from being released into the bloodstream and reduces the creatinine adsorption capacity of the zeolites in the fiber; however, their adsorption capacity is still 67% of the free zeolites [36].

Currently, hemodialysis has been found to reduce only 66–75% urea in the blood. Bahramimehr et al. applied two different nano electrospinning fibers (hybrid and coaxial) to reduce creatinine and urea in the blood of dialysis patients [86]. Hybrid nanofibers were made of zeolite 940-HOA (beta), Fe_3_O_4_, polyacrylonitrile (PNA), nettle plant’s leaf extract and a coat of urease enzyme (PAN-magnetic-zeolite-nettle extract coating urease enzyme, PMZNU). The other nanofibers were fabricated by core-shell electrospinning and contained cellulose acetate phthalate (CAP) and polyurethane (PU); however, the CAP-PU fiber cannot reduce creatinine as expected, and 3 h is the maximum time required to reduce creatinine. Bahramimehr et al. then compared the two nanofibers and found that the tolerance of PMZNU against temperature and rupture is more preferable to that of CAP-PU filter. Moreover, they observed that the surface of CAP-PU membrane can be loaded with additional enzyme and drugs, which could be useful for dialysis patients [86].

**Fig. 14:**
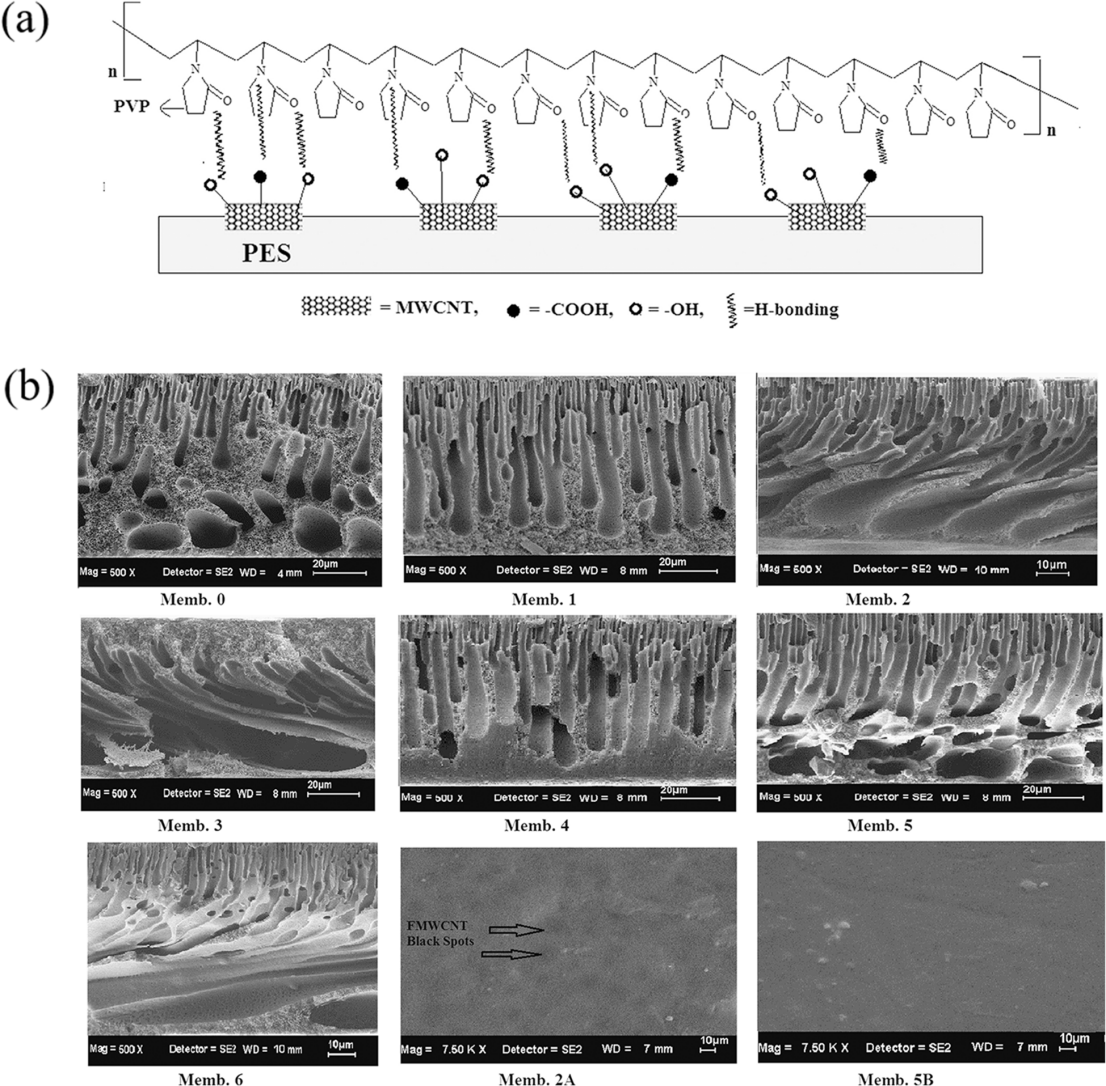
**a** Schematic representation of PES/f-MWCNT/PVP nano-hybrid membrane. **b** Cross-sectional and surface SEM micrograph of PES and PES nano-hybrid HD membranes. Memb. 2A and 5B represent surface topology of membrane 2 and 5. In Memb. 2A, black spots indicate the presence of FMWCNT. Reproduced with permission [87]. Copyright 2014, Elsevier

Another kind of nano-hybrid membrane was designed by Irfan et al. They initially synthesized nano-composites by mixing up acid functionalized multiwall carbon nanotubes (f-MWCNT) and PVP in dimethylformamide (DFM), and then mixed these with PES to fabricate PES/PVP-f-MWCNT nano-hybrid hemodialysis membranes [87]. The schematic representation of this process is presented in Fig. 14. Multiwall carbon nanotubes (MWCNT) are hydrophobic, but they can be easily modified and attached to different functional groups. Acid treatment of MWCNT makes it dual in nature, where they develop some hydrophilic parts, like -COOH and -OH [88, 89]. Its heredity carbon part creates sites for attachment of hydrophobic polymers by hydrophobic–hydrophobic interaction and π – π stacking, while the acid and hydroxyl groups on the other side attract the hydrophilic component by hydrogen bonding, dipole–dipole interaction and dispersion forces [90]. This new membrane has been shown to enhance antifouling properties and can reduce more than 50% of the urea and creatinine in a 4 h dialysis, while the reduction of pristine PES was less than 10% [87].

In addition to creatinine and urea, excess water should be removed through dialysis treatment. Tsuge et al. developed a poly (sodium acrylate) (PSA) nanofiber mesh, which has the potential to be utilized as a new filter in a wearable blood purification system [91]. The strongly hydrophilic property of PSA is attributed to its pendent carboxylate anions. When the PSA nanofiber meshes with a hydrophilic sodium carboxylate group, it exhibits a specific surface area 393 times greater than its corresponding film, and can remove excess fluids from solution and blood. However, PSA, itself, is not an ideal biocompatible material and cannot adsorb other uremic toxins.

#### Nanoparticles

Nanoparticles (NPs) are a wide class of materials with at least one dimension less than 100 nm and are composed of three layers, including a surface layer, shell layer and core [92]. The surface layer may be functionalized with a variety of substances [92].. NPs, in contrast to their microparticles or bulk materials, are suitable candidates for various applications due to their high surface area, mechanical properties and magnetic properties [92, 93]. These applications include drug delivery, tumor detection, cell labeling and other biological applications [92, 93]. They also can be applied in water decontamination due to their magnetic properties, which, effectively, dominates when the size of nanoparticle is less than the critical value (i.e., 10–20 nm). It has been demonstrated that magnetically assisted hemodialysis exhibits a greater overall removal efficiency than conventional hemodialysis [94]. Sufficient control over the specific morphology, size and magnetic properties of NPs is vital for enhancing material removal [92]. Moreover, a significant number of studies have demonstrated a promising role of nanoparticles in the diagnosis and treatment of acute kidney injury (AKI) and chronic kidney disease [93, 95]. For instance, oxidative stress has been confirmed to be a predominant pathogenesis for AKI, but the clinical efficacy of common antioxidants currently is limited due to lack of targeting ability [93]. In this case, antioxidant nanoparticles could be used in reactive oxygen species-targeted therapy, because they can target oxidative stress in renal mitochondria [93]. Furthermore, NPs play an important role in deliver of drugs and nucleic acids by means of serving as a kidney-targeting transport system. Upon ESKD, nanotechnology could improve efficacy of hemodialysis and reduce side effects, such as dialysis-induced oxidative stress, protein adsorption and plate adhesion [95]. The primary goal of hemodialysis is replacing kidney excretory function to restore the intracellular and extracellular fluid environment, which represents the transfer of various proteins or electrolytes, and therefore is not limited to uremic toxins. Blood purification technology is also widely used in the treatment of liver failure, poisoning, and immune-related diseases. It is also necessary to develop some biomaterials that can effectively eliminate molecules related to cellular dysfunction, such as iron ions and ROS.

Urea is not only the most abundant uremic toxin constituent in blood, it is also difficult to remove [96]. For instance, it has been reported that activated carbon has a very poor affinity towards urea [96]. Oxidized starch has been used for urea removal for more than 30 years, and more recently, Abidin et al. developed an oxidized starch nanoparticles (oxy-SNPs) for urea adsorption via chemical dissolution, non-solvent precipitation and liquid phase oxidation [97]. These nanoscale oxy-SNPs have a maximum adsorption capacity of 185.2 mg/g [97]. The urea molecules are deposited on the free surface of oxy-SNPs through chemical bonding, where the oxidation of starch introduces carbonyl groups that each provide one active site for the amine group of urea; however, not all the sites are accessible due to steric hindrance.

**Fig. 15:**
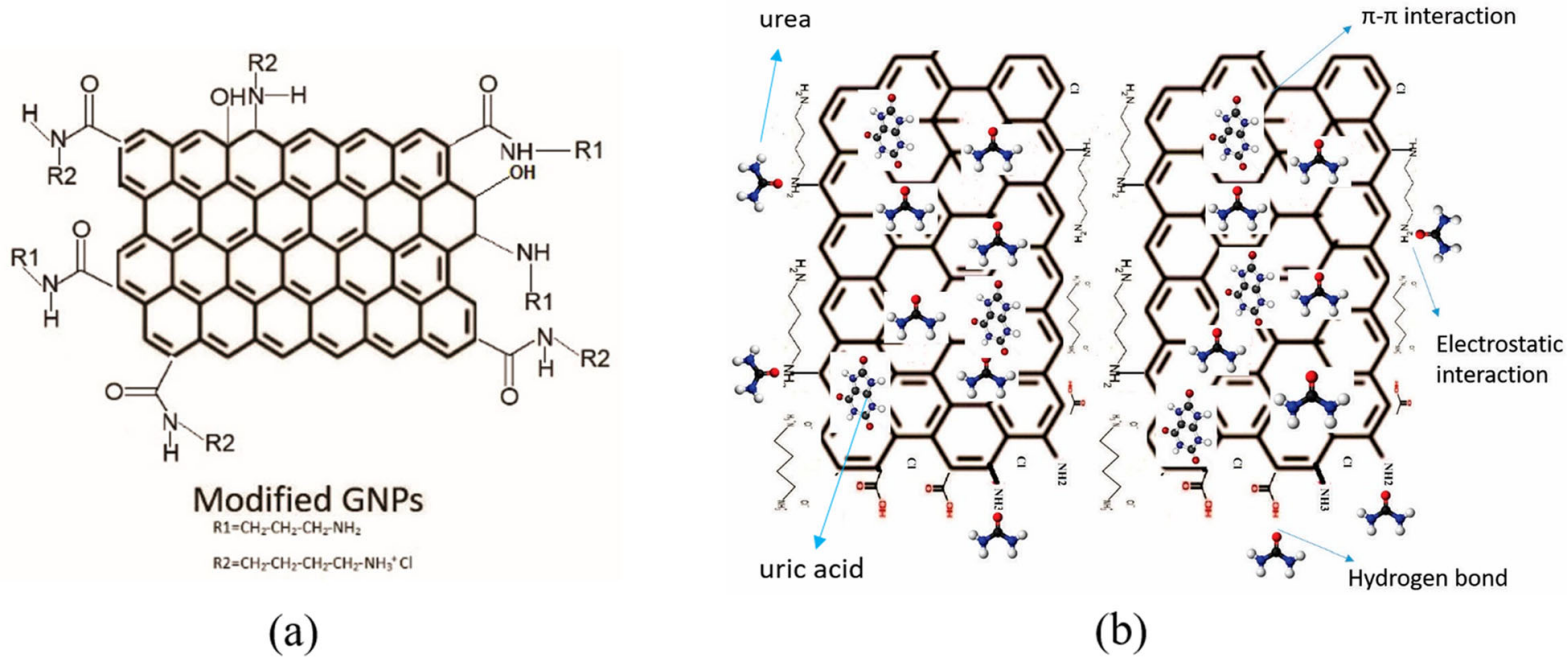
**a** Structure of modified GNPs and **b** proposed scheme for urea and uric acid adsorption onto the modified GNPs. Reproduced with permission [98]. Copyright 2019, MDPI

According to previous studies, carbon-based nanostructures are excellent for adsorbing uric acid. A fabrication that uses ultrasound waves has been introduced by Cabello-Alvarado et al. and involves the modification of graphene nanoplatelets (GNPs) with amines [98]. These modified GNPs can achieve the maximum percentage of removal of 97% urea and uric acid, which is mostly ascribed to the presence of amino groups with covalent bond between carbon and nitrogen that help to promote selective adsorption. The structure of modified GNPs, as well as the proposed scheme for urea and uric acid adsorption onto the modified GNPs are shown in Fig. 15. On the other hand, carbon black possesses high microporosity and low cost compared to other carbonaceous nanomaterials. Andrade-Guel et al. synthesized Nylon 6/modified carbon black nanocomposites using an ultrasound-assisted melt-extrusion method, where they initially modified carbon black with citric acid using variable-frequency ultrasound and then incorporated it into a polymeric matrix of Nylon 6 [99]. The modified carbon black, as well as modified carbon black nanocomposites show superior uric acid removal (78–82%) and hemocompatibility (1.6–1.8% hemolysis).

Recently, ceria NPs have been reported to possess robust scavenging capability for multiple ROS, and the cerium (IV) sites are accountable for the oxidation of H_2_O_2_, while the cerium (III) sites are known to remove ⋅OH and O_2_⋅^-^ [100–102]. Ni et al. demonstrated that ceria NPs could protect against hepatic ischemia reperfusion injury in the early stage when it just starts [103]. They found that ceria NPs likely co-localize in Kupffer cells and liver sinusoidal endothelia cells, where they can directly scavenge ROS.

**Fig. 16:**
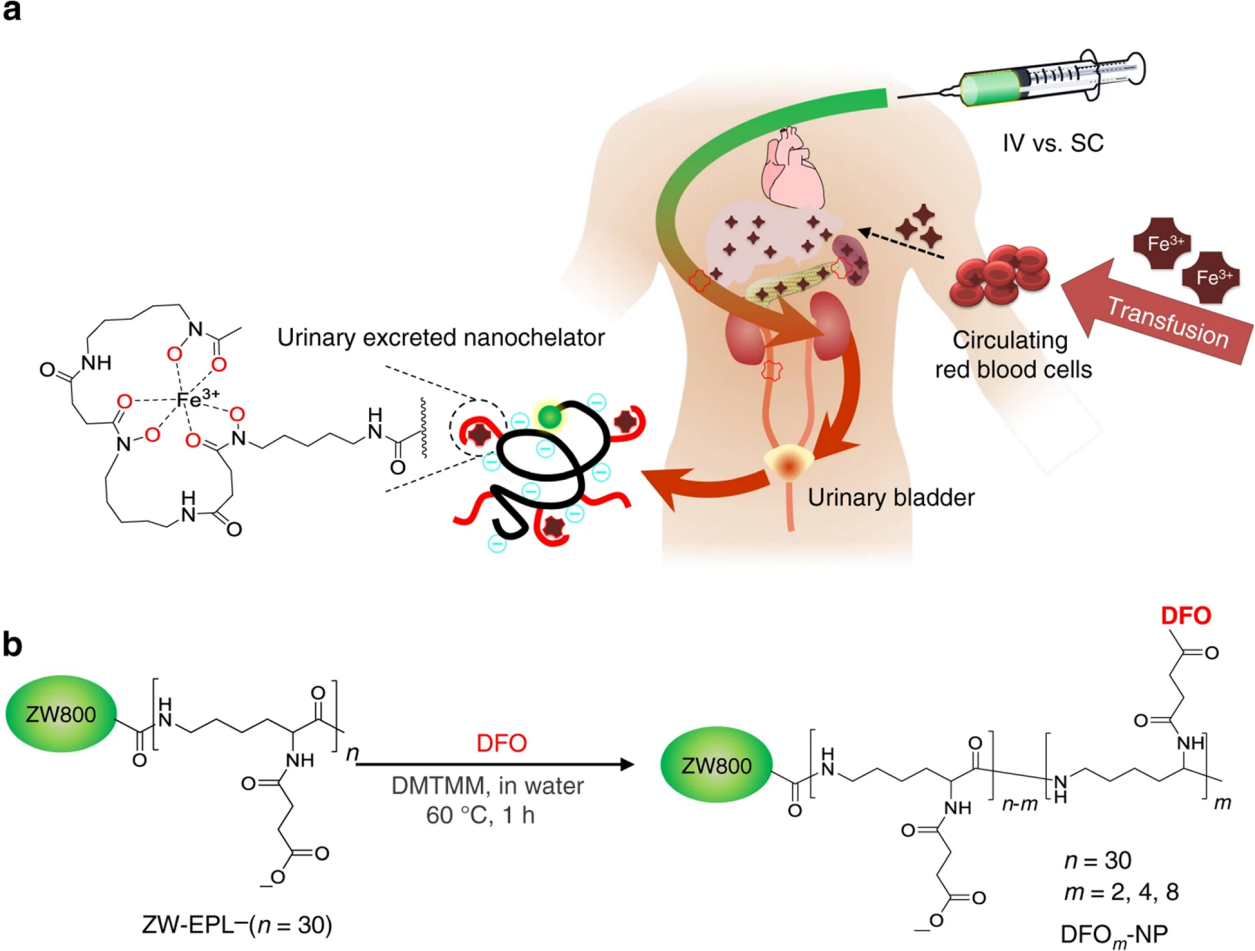
Renal clearable nanochelators to treat secondary hemochromatosis. **a** Nanochelators are composed of multiple DFO moieties conjugated on renal clearable EPL backbone to minimize nonspecific uptake into immune-related organs, while efficiently capturing plasma iron and being cleared exclusively by the kidneys. **b** Synthetic scheme of renal clearable DFO-NPs. m represents the number of DFO moieties conjugated on EPL. NIR fluorescent ZW800-1C (green color) was conjugated to track the fate of nanochelators in the body. Reproduced with permission [106]. Copyright 2019, Springer Nature

Iron is an essential metal nutrient, but iron-overload can cause progressive and sometimes irreversible end-organ injury before clinical symptoms develop [104]. There is still a need for novel drug candidates for patients with iron-overload because iron chelators have their own drug toxicity and limited long-term efficacy [105]. Kang et al. designed and synthesized the renal clearable nanochelator as an enhanced efficacy and safety iron chelation therapy [106]. They used ε-poly- L –lysine (EPL), a natural anti-microbial cationic peptide, as a biocompatible backbone, where the primary amines on EPL were converted to carboxylates before conjugation of deferoxamine (DFO) moieties on the backbone. DFO serves as the iron-binding domain in the nanochelator and contains three hydroxamate groups that bind iron at 1:1 stoichiometry [107], and thus prevents iron ions from catalyzing hydroxyl radical formation through complete chelation of iron that leaves it without unoccupied coordination sites [105]. DFO-NPs have been shown to provide favorable bio-distribution, pharmacokinetics and pharmacodynamics when compared to native DFO, as well as to significantly reduce kidney damage caused by iron overload without demonstrating their own nephrotoxicity (Fig. 16) [106].

Bilirubin is one of the metabolites of hemoglobin that accumulates in blood. In patients who lack the ability to remove extra bilirubin, such as acute hepatic failure, the concentration of bilirubin will rise and exert negative effects on the central nervous system [108]. Peng et al. prepared Kevlar nanofiber-based porous beads, a promising material that exhibits efficient and effective removal of both bilirubin and creatinine, which is due to their porous structure and associated large surface area [109]. They dissolved Kevlar fiber in DMSO/KOH to prepare Kevlar nanofiber before dropping in ethanol to fabricate beads, where CNT was then incorporated into beads to further increase the adsorption capacity. Owing to the favorable hydrophilicity of Kevlar nanofibers, the beads exhibit excellent blood compatibility in terms of a low hemolysis ratio, suppressed platelet and complement activation, as well as a prolonged clotting time [109, 110]. Furthermore, the endothelial cell culture results revealed that the beads demonstrate low cytotoxicity.

## The toxicity of biomaterials for blood purification

Biomaterials have been widely employed in medical fields, such as cell and tissue cultures, artificial organs, and devices used for blood purification. High biocompatibility is required to avoid the side effects, which is especially true for blood-contacting devices [109, 111]. A series of blood responses are triggered as soon as blood comes into contact with a surface without hemocompatibility, such as hemolysis, plasma coagulation, platelet and complement activation, which then lead to blood coagulation and thrombus formation. Among the responses, protein adsorption occurs at the interface immediately and plays a vital role in material-associated thrombus formation. These reactions during blood purification are harmful or even lethal for patients [112, 113]. During clinical hemodialysis, the most commonly used way to prevent blood coagulation is the injection of anti-coagulant reagents, like heparin. However, the use of heparin may cause side effects in patients, leading to spontaneous hemorrhage, osteoporosis and allergic reactions [114]. Two main strategies have been developed to address this issue, membrane modification and anticoagulant modification. The charge, functional group, topology, and specifically the hydrophilicity of the surface could affect blood compatibility [115–117]. It has been found that materials with hydrophilic surfaces exhibit resistance to protein adsorption and platelet adhesion, which may endow the bio-interface with a favorable anti-fouling property [109, 118–120]. Moreover, it has been reported that the MMM blood compatibility is comparable to reference commercial dialysis fibers that are currently used in the clinic [71]. Adding additives in the production of dialysis membranes is also a practical strategy. Modi et al. synthesized hydrophilic zeolitic imidazole framework decorated graphene oxide nanosheets (ZGs) that remarkably improves biocompatibility and separation performance, as well as possessed antioxidant and hemocompatibility properties when used as additives (0–1 wt%) in PES hollow fiber membranes [121]. Iron oxide nanoparticle (Fe_2_O_3_ NP) is another alternative additive. Said et al. developed membranes made up of polysulfone and Fe_2_O_3_ NPs, and found that these membranes have improved biocompatibility and excellent clearance of urea and lysozyme [122].

**Fig. 17:**
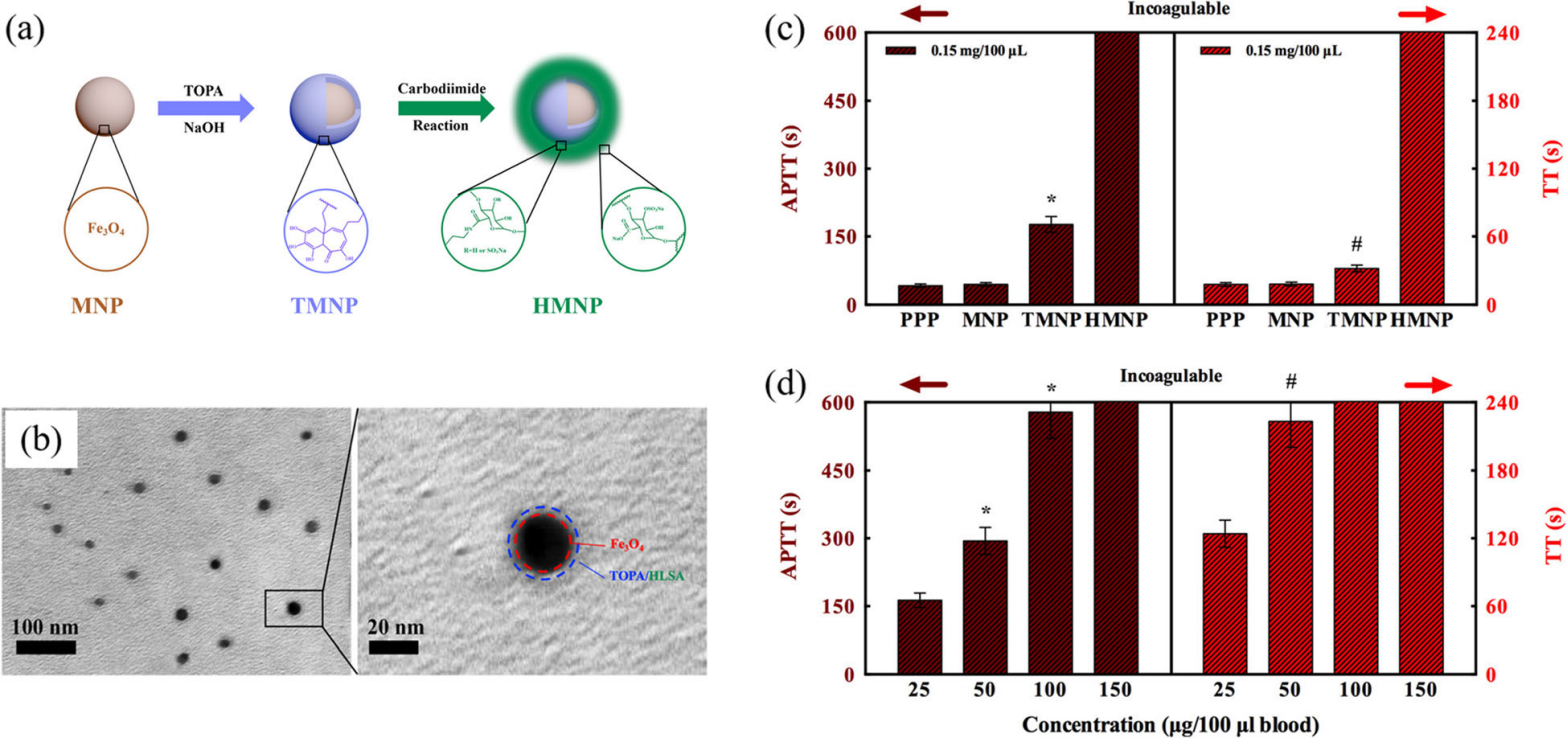
**a** Scheme for preparation of TMNP and HMNP. **b** TEM images of HMNP. **c** Activated partial thromboplastin time (APTT) and thrombin time tests (TT) for MNP, TOPA modified MNP (TMNP) and HMNP at a concentration of 150 μg/100 μL blood. For the control group (PPP), 5 μL PBS was added instead. **d** APTT and TT for HMNP at concentrations of 25, 50, 100 and 150 μg/100 μL blood. Reproduced with permission [123]. Copyright 2020, American Chemical Society

Additionally, self-coagulant membranes are a potential way forward for the design of an advanced biomedical dialyzer. It has been reported that integration of an anti-coagulant reagent, such as heparin, into the biomedical dialysis membrane is a workable way to prevent coagulation during hemodialysis, and thus reduce the dose of anticoagulant reagent [116]. In view of the anticoagulant activity of heparin that is partially ascribed to its ionic groups, it is reasonable to achieve improved hemocompatibility of biomaterial by introducing multiple heparin-mimetic groups, such as sulfonic (−SO_3_Na), carboxylic (−COONa) and hydroxyl (−OH) groups, to the surface (Fig. 17) [123]. The heparin-like/-mimicking polymer modified membranes also possess excellent blood and cell compatibilities that are comparable to heparin immobilized membranes [124]. In addition, the heparin-mimicking polymer has the advantages of tunable chemical structure, low cost, long-term activity and biological degradable stability [125–129]. Synthesized heparin-mimetic magnetic nanoparticle (HMNP) is another suitable biomaterial that combines iron oxide and heparin. HMNP appears to be a promising alternative given that it can be used as an anticoagulant reagent in long-term hemodialysis. Furthermore, all components of it are biocompatible, and it possesses outstanding recycle stability, where it can be collected by a magnetic field after hemodialysis [123]. Wang et al. fabricated HMNP through immobilizing heparin-mimetic sodium alginate (HLSA) on the surface of iron oxide magnetic nanoparticles (MNPs) by using 3,4,5-trihydroxyphenylalanine (TOPA) as a biological adhesive (Fig. 17) [123]. Thus, these membranes possess a heparin-like structure, as well as favorable anti-fouling properties, which make them ideal biomedical materials for avoiding toxicity during blood purification.

## Conclusion

In conclusion, we summarized the novel biomaterial for blood purification that has been reported in recent years. These biomaterials were roughly divided into 3 categories, including adsorbents, polymeric composite membranes and nanomaterials. Adsorbents, such as zeolites, activated carbon and CTNs, are commonly incorporated inside polymer materials to enhanced ultrafiltration capacity. When adsorptive particles are dispersed throughout the polymer matrix, removal efficiency of uremic toxins can be notably improved by combining adsorption and diffusion of uremic retention solutes, which has been validated in studies related to MMM and f-CNT/PES composite membranes. Due to large surface area and porosity, nanomaterials and MOFs both exhibit favorable adsorption abilities. In addition, the fabrication of polymeric nanofibers is cost-effective and Zr-based MOFs have good reusability.

Permeation is a vital property of a membrane used for hemodiafiltration, since low removal rate of toxins makes the patients suffer through longer periods of dialysis that are higher in expenditure. When the concentration of free toxin on the dialysate side is maintained at low levels, there is a continuous diffusion driving force over the whole hemodialyzer length, especially for PBUTs [130, 131]. As mentioned in this paper, materials with large surface area and porosity are promising candidates for producing hemodialysis membrane. Embedding adsorptive particles in a polymer membrane matrix is also an efficacious method for achieving a more efficient clearance rate of uremic toxins, which is owing to the maintenance of a concentration difference. There are also a few adsorbents whose adsorption ability is less correlated with pore structure, such as NPCA beads. Therefore, it is crucial to analyze the respective molecular structure of adsorbent and uremic toxins, as well as the interaction within them.

Dialysis is a long-term and life-sustaining treatment for ESKD patients, and nearly 500 L of tap water is needed to obtain pure dialysis water for a single hemodialysis session, which is high in both cost and energy consumption [132]. Therefore, the simple and low-cost manufacture method of dialysis material is of great significance for its clinical application. In addition, microbiological biofilm caused by water stagnation will still form when the tubing system is inadequate or there is improper machine maintenance [133, 134]. The bacterial growth and lysis in the water purification system, and thus pyrogens could be transferred into the blood of patients, which causes micro-inflammatory status, leading to cardiovascular side effects or acute side effects, like fever and muscular cramps [133–139]. Several sorbent systems for endotoxins (e.g., lipopolysaccharide) removal have been reported, such as functionalized nanoparticles [140], activated carbon [26, 141], addition of a PS-poly (ethylene glycol) copolymer (PS-PEG) and bleach sterilization [133]. Additionally, the MMM offers superior endotoxin removal and acts as a safe barrier that avoids inflammatory responses without decreases in uremic toxins removal [71]. In general, biomaterials with long-term and high ultrafiltration, favorable biocompatibility and cost-effective fabrication methods are ideal candidates for blood purification. Even if the new biomaterials mentioned in this review have improved adsorption capacity and biocompatibility, there is still much work to be done that focuses on the modification of materials so that they are suitable for clinical application.

## Data Availability

Not applicable.

## Abbreviations

ESKD: End stage kidney disease
PBUTs: Protein-bound uremic toxins
ROS: Reactive oxygen species
PCS: p-cresyl sulfate
IS: Indoxyl sulfate
CMPF: 3-Carboxy-4-methyl-5-propyl-2-furanpropionic acid
IAA: Indole-3-acetic acid
OATs: Organic anion transporters
HA: Hippuric acid
pCG: p-cresyl glucuronide
AC: Activated carbon
CNTs: Carbon nanotubes
NPCA: Nitrogen-containing porous carbon adsorbent
MFI: Zeolite silicalite
MOR: Mordenite
STIs: Ion-exchanged stilbites
MOF: Metal-organic framework
HCl: Hydrochloric acid
MIPIOPs: Molecular-imprinted polymer inverse opal particles
SCCBs: Silica colloidal crystal beads
GelMA: Methacrylate gelatin
PEGDA: Polyethylene glycol diacrylate
CDs: Cyclodextrins
PCD: Poly-β-cyclodextrins
ECH: Epichlorohydrin
PES: Polyethersulfone
PVP: Polyvinylpyrollidone
PAN: Polyacrylonitrile
MMMs: Mixed-matrix membranes
TAT: Thrombin-antithrombin III-complex
SS: Styrene sulfonate
EGMA: (ethylene glycol) methyl ether methacrylate
f-CNTs: Multiwall CNTs
EVOH: Ethylene-co-vinyl alcohol
HFIP: 1,1,1,3,3,3-hexafluoroisopropanol
PNA: Polyacrylonitrile
PMZNU: PAN-magnetic-zeolite-nettle extract coating urease enzyme
CAP: Cellulose acetate phthalate
PU: Polyurethane
f-MWCNT: Functionalized multiwall carbon nanotubes
DFM: Dimethylformamide
PSA: Poly (sodium acrylate)
NPs: Nanoparticles
AKI: Acute kidney injury
oxy-SNPs: Oxidized starch nanoparticles
GNPs: Graphene nanoplatelets
EPL: ε-poly- L –lysine
DFO: Deferoxamine
ZGs: hydrophilic zeolitic imidazole framework decorated graphene oxide nanosheets
HMNP: Heparin-mimetic magnetic nanoparticle
HLSA: Heparin-mimetic sodium alginate
MNPs: Magnetic nanoparticles
TOPA: 3,4,5-trihydroxyphenylalanine
APTT: Activated partial thromboplastin time
TT: Thrombin time tests
PS-PEG: PS-poly (ethylene glycol) copolymer
